# Computational analysis of the flexibility in the disordered linker region connecting LIM domains in cysteine–glycine-rich protein

**DOI:** 10.3389/fgene.2023.1134509

**Published:** 2023-03-29

**Authors:** Pankaj Kumar Chauhan, R. Sowdhamini

**Affiliations:** ^1^ National Centre for Biological Sciences Tata Institute of Fundamental Research, Bangalore Karnataka, India; ^2^ Molecular Biophysics Unit, Indian Institute of Science, Bangalore, India; ^3^ Institute of Bioinformatics and Applied Biotechnology, Bangalore, India

**Keywords:** CSRP3, disordered region, cardiomyopathy, PPI, molecular dynamics

## Abstract

One of the key proteins that are present in the Z-disc of cardiac tissues, CSRP3, has been implicated in dilated and hypertrophic cardiomyopathy leading to heart failure. Although multiple cardiomyopathy-related mutations have been reported to reside on the two LIM domains and the disordered regions connecting the domains in this protein, the exact role of the disordered linker region is not clear. The linker harbors a few post-translational modification sites and is expected to be a regulatory site. We have carried out evolutionary studies on 5614 homologs spanning across taxa. We also performed molecular dynamics simulations of full-length CSRP3 to show that the length variations and conformational flexibility of the disordered linker could provide additional levels of functional modulation. Finally, we show that the CSRP3 homologs with widely different lengths of the linker regions could display diversity in their functional specifications. The present study provides a useful perspective to our understanding of the evolution of the disordered region between CSRP3 LIM domains.

## 1 Introduction

The cysteine and glycine-rich protein (CSRP) gene belongs to a family of proteins, vitally involved in basic processes such as differentiation, growth, and gene regulation (Weiskirchen et al., 1995). Three types of this protein, CSRP1, CSRP2, and CSRP3, have been noted in three genomic locations in the human genome: chromosome 1, 10, and 11, respectively. CSRP1 is implicated in stress response by binding to zyxin (Schmeichel and Beckerle, 1998), while CSRP2 is implicated in leukemia (Wang et al., 2017).

The gene CSRP3, or muscle LIM protein (MLP) (HGNC:24722; chr. location: 11p15.1), is organized into six exons encoding a 194 amino acid long protein (Fung et al., 1996; Knöll et al., 2002). CRSP3 protein contains two LIM domains connected by a disordered region rich in glycyl and prolyl residues and is highly flexible. Each LIM domain, in turn, possesses two Zn-finger subdomains, each one having specific binding partners including transcription factors such as MyoD, MRF4, GATA4, and SRF (Buyandelger et al., 2011). The LIM domains are also involved in a distinct functional role such as the regulation of the expression of target genes. Alternatively spliced transcript variants with different 5′ UTR, but encoding the same protein, have been found for this gene.

CSRP3 is primarily expressed in the heart and to a lesser extent in prostate tissue of humans. The function of CSRP3 is attributed as a mechanical stretch sensor in the Z-disc complex of the heart (Buyandelger et al., 2011). A significant reduction in CSRP3 protein levels was reported in dilated and ischemic cardiomyopathy patients leading to heart failure (Zolk et al., 2000). In addition, W4R or C58G point mutations in this gene implicated hypertrophic cardiomyopathy and heart failure (Knöll et al., 2002; Knoll et al., 2010; Ehsan et al., 2018). Hence, mutations in this gene are thought to cause heritable forms of hypertrophic cardiomyopathy (HCM) and dilated cardiomyopathy (DCM) in humans. As of today, close to 20 mutations have been realized on CSRP3 and most of them are found to have deleterious effects. We had recently conducted a virtual saturation mutagenesis to understand the structural effects of amino acid residues and positions (Chauhan and Sowdhamini, 2022). Here, we also showed that C-terminal LIM exhibits higher conservation compared to N-terminal LIM. A study by Hoffmann *et al.* demonstrated that N-terminal LIM is involved in self-association of CSRP3 while C-terminal LIM exhibits direct interaction with actin filaments (AFs) and stabilizes AFs, cross-linking them into bundles (Hoffmann et al., 2014). This suggests that C-terminal LIM is relatively more important in AF stability. CSRP3 oligomerizes *via* the N-terminal LIM domain that seems to be initiated by post-translational modification, in particular the O-glycosylation, as shown in a study by Chiaki Nagai-Okatani and Naoto Minamino (Nagai-Okatani ¤a and Minamino, 2016). The study also postulates six O-glycosylation sites in the linker region between two LIM domains. The linker region may regulate the protein–protein interaction (PPI). It was demonstrated that the linker region between amino acids 94 to 105 interacts with cofilin 2 (CFL2) (Papalouka et al., 2009). It necessitates a thorough examination of the linker region in homologs to understand its role and evolution.

In this work, we have examined the evolutionary landscape of the CSRP3 gene in other species and report various aspects, such as length variations of the two LIM domains and the connecting linker region. To the best of our knowledge, this is the first study which explores the linker region variation and its taxonomic distribution. Few of the homologs which exhibit anomalous linker lengths have been discussed. All-atom long-length molecular dynamics simulations of the full-length CSRP3 protein reveal spontaneous interactions between the two LIM domains, facilitated by the flexible linker region. The backbone conformational flexibility of the linker region has been investigated. In order to understand the functional variations, we followed the post-translational and protein–protein interaction patterns of human CSRP3 and few homologs of variable lengths to show that such length variations could provide functional versatility in terms of their choice of partner proteins.

## 2 Materials and methods

### 2.1 Sequence retrieval and analysis

The query term “CSRP3” was searched in the NCBI protein database (https://www.ncbi.nlm.nih.gov/protein/), and the human CSRP3 protein [accession number: NP_003467.1] FASTA sequence was selected. To obtain CSRP3 protein homologs from different organisms, the NCBI domain enhanced lookup time accelerated BLAST (DELTA-BLAST) (Boratyn et al., 2012) was performed where the previously obtained sequence was used as the query input and nr protein was selected as the database. The DELTA-BLAST parameters of Expect threshold = 0.00005, Matrix = BLOSUM62, gap costs = Existence:13 Gap: 1, compositional adjustments = composition-based statistics, PSI-BLAST threshold = 0.00005, DELTA-BLAST threshold = 0.00005, pseudocount = 0, and max output hits = 20000 were set, and it was run for one iteration. Next, the filter of query coverage >75% and percent identity >30% was applied to the output and sequence IDs were downloaded. The efetch mode of Entrez Programming Utilities (E-utilities) (Rédei, 2008) was utilized to retrieve protein sequences for these IDs programmatically. The domain prediction in these sequences was carried out using the HMMSCAN module of HMMER-3.1 (Eddy, 2008) on the all domains hmm of the PFAM dataset (Mistry et al., 2021). Sequences having query coverage <75% full-length human CSRP3 protein were filtered that were missed during blast filtering, and also, a list of dual-LIM, multi-LIM, and other domain containing IDs was created. The disordered region from dual-LIM sequences was extracted based on LIM1 and LIM2 boundaries. The histogram of length variability in LIM domains and the disordered region across sequences was plotted. The amino acid propensity of the disordered region was calculated as follows:

$$P_{AA_{i}}=\frac{\sum_{D=1}^{D=N}Count\left(AA_{i}\right)/\sum_{j=1}^{N}Length\left(D_{j}\right)}{\sum_{L_{1}+D+L_{2}=1}^{L_{1}+D+L_{2}=N}Count\left(AA_{i}\right)/\sum_{j=1}^{N}Length\left(L_{1j}+D_{j}+L_{2j}\right)}.$$

The $P_{AA_{i}}$ is the propensity of an amino acid $AA_{i}$ , N is total number of sequences, $D_{j}$ is the disordered region of the j th sequence, $L_{1j}$ is the first LIM of the j th sequence, and $L_{2j}$ depicts the second LIM of the j th sequence.

### 2.2 Homology modeling of human CSRP3 protein and *Arabidopsis* and nematode representative homologs

The protein sequence of human CSRP3 was used for homology-based structure modeling. Next, in order to find a suitable template for modeling, protein BLAST (Camacho et al., 2009) was utilized to search for the nearest structural homolog in the Protein Data Bank (PDB) (Berman et al., 2003). Blast PDB hits, namely, 2O10 (human CSRP3 LIM1), 2O13 (human CSRP3 LIM2), and 1B8T (chicken CSRP1) NMR structures, were used for multi-template modeling of human CSRP3 full-length protein. The first conformer from each structure was used in the template selection. A homology-based structure modeling tool, MODELLER 9.12 (Eswar et al., 2006), was used for CSRP3 structure modeling. Ten homology models were generated and ranked according to the DOPE score. Then, the top five ranked models were assessed for structure validations using SAVES 5.0 (Laskowski et al., 1993) (https://servicesn.mbi.ucla.edu/SAVES/) and the ProSA server (Wiederstein and Sippl, 2007). The best-predicted model assessed based on the DOPE score, Ramachandran plot, and ProSA profile was selected for further structural analysis. In the case of homologs in *Arabidopsis* and nematode genomes, where CSRP3 contains short-length and long-length linker regions, respectively, a similar strategy was adopted. The details of query coverage, percent identity, and templates used in each sequence modeling are provided in Supplementary Table S1.

### 2.3 Molecular dynamics simulations of model structures

The best-predicted model was minimized at pH 7 using PROPKA from Protein Preparation Wizard in the Maestro package (Schrödinger Release 2020: Maestro, Schrödinger, LLC, New York, NY, 2020). Next, the structure was restrain-minimized using the OPLS3e force field (Harder et al., 2016) and solvated with the TIP3P water system using the System Builder from the Desmond module (Bowers et al., 2006). To account for periodic boundary conditions, the orthorhombic box was used and its volume was minimized by having a buffer distance of 10Å. The aforementioned system was neutralized with 15 CL^-^ ions, and additional 150 mM NaCl salt was added. The OPLS3e force field was chosen as the force field, and Molecular Dynamics (MD) run was carried out in the Molecular Dynamics package on the output generated by the System Builder. In the MD run, a short relaxation run was initiated with the default protocol on the solvated system generated from previous steps. After relaxation, the actual production MD simulation was accomplished under NPT constraint parameters and the OPLS3e force field. In the simulation, the default settings of the RESPA integrator (Humphreys et al., 1994) (2 femtoseconds time step for bonded or near non-bonded interactions and 6 femtoseconds for far non-bonded interactions) were incorporated. The temperature was kept fixed at 300 K using a nose-Hoover thermostat algorithm (Martyna et al., 1992). Similarly, the pressure (1 bar) was fixed using the Martyna–Tobias–Klein method (Martyna et al., 1994). The production MD simulation was executed for 200 nanoseconds in triple replicates. This protocol was followed for human CSRP3 and homologs from *Arabidopsis* and nematode model structures as representative for trimodal distribution of the linker region.

### 2.4 MD simulation event analysis

The simulation interaction diagram (SID) and simulation event analysis (SEA) modules of the Desmond package were explored for the MD trajectories analyses. The root mean square distance (RMSD) and root mean structure fluctuation (RMSF) of the protein backbone were calculated for the entire duration of simulation time by aligning them to the reference frame (0th frame) in the SID. The same protocol was followed for the replicates. Similarly, the Radius of Gyration (ROG) and energy change were calculated for each trajectory using the SEA module. The distance density map showing the distance between LIM1 and LIM2 throughout the simulation point was created using the proxy distance between Cys 10–Phe 176 for human CSRP3 and similarly for the *Arabidopsis* and nematode representative. Next, the phi–psi values corresponding to the disordered region were calculated for the structure at 0th frame (0 ns), 500th frame (100 ns), and 1000th frame (200 ns), and an arrow map was generated to highlight the deviation in phi–psi values at these stages. Furthermore, the time-dependent evolution of secondary structure elements (SSEs) along the MD simulation trajectory was generated by the STRIDE package (Frishman and Argos, 1995) in VMD software (Humphrey et al., 1996).

### 2.5 Post-translational modifications and taxonomy

The post-translational modifications (PTMs) in the disordered region of protein sequences were predicted using the MusiteDeep online server (https://www.musite.net/) (Wang et al., 2020). The PTMs such as phosphorylation, glycosylation, ubiquitination, SUMOylation, acetylation, methylation, palmitoylation, and hydroxylation were considered and score cut-off of 0.8 and 0.9 were explored. The frequency of each PTM was calculated across the disordered sequences. The taxonomic distribution of sequences was analyzed at the phylum level using ETE toolkit (Huerta-Cepas et al., 2016).

### 2.6 Protein–protein interactions

The protein–protein interaction analysis was executed on the STRING database server (Szklarczyk et al., 2021). Protein sequences for protein IDs NP_001180500.1 (human CSRP1), NP_001400464.1 (human CSRP2), NP_003467.1 (human CSRP3), TWW71823.1 (sansaifugu CSRP2), KRX47685.1 (Trichinella CSRP2), CAB71053.1 (*Arabidopsis* LIM), and PIN15380.1 (*Handroanthus* MLP) were queried for protein–protein interactions. In case of no match, the next most similar (by % identity) sequence suggested by the server was used as a proxy for PPI.

## 3 Results

### 3.1 CSRP3 homolog sequence analysis

**FIGURE 1:**
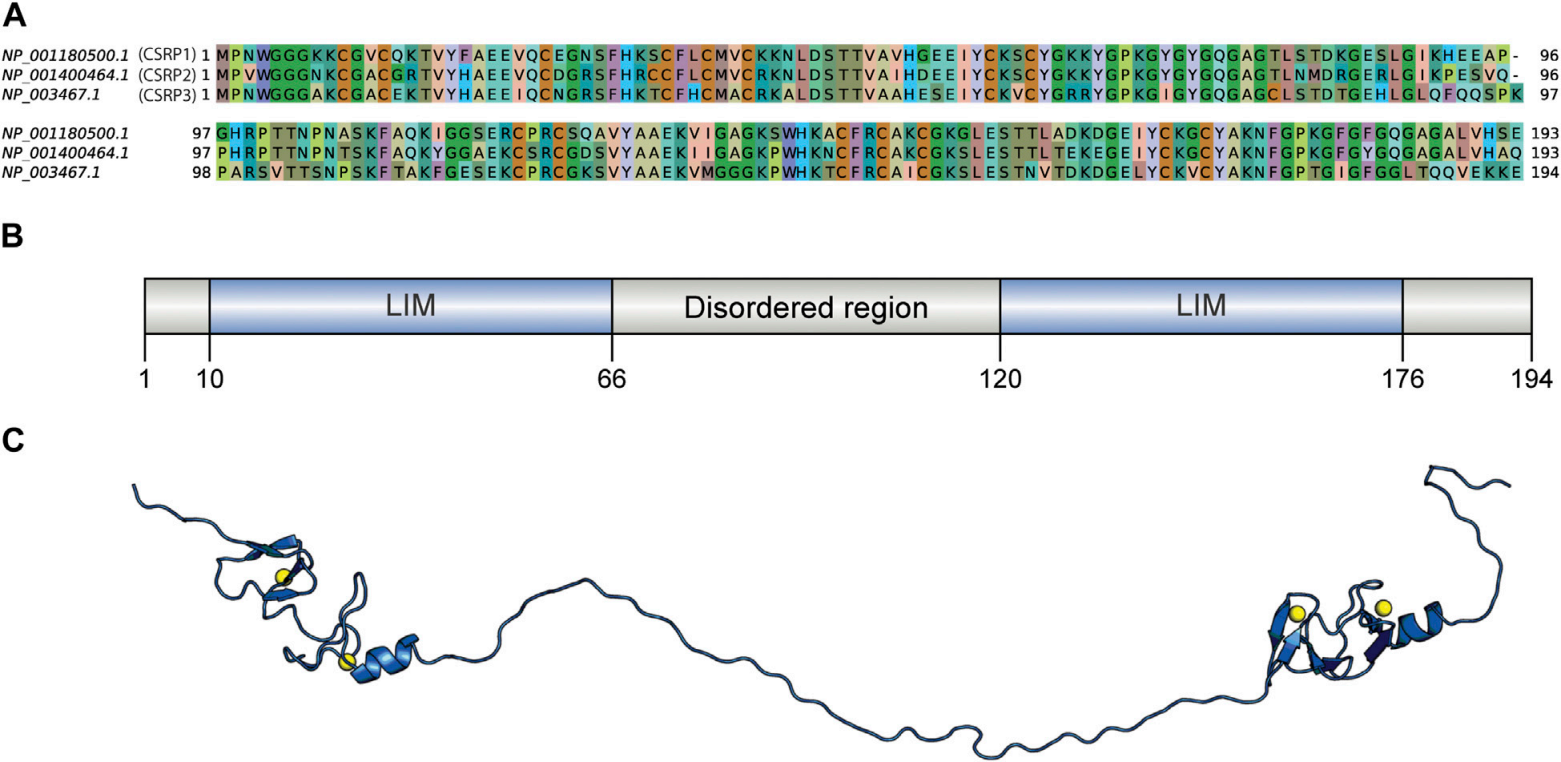
Sequence comparison of human CSRPs homologs and the CSRP3 model structure. **(A)** Sequence alignment of CSRP1, CSRP2, and CSRP3 proteins. **(B)** Domain architecture of human CSRP3 as predicted by the PFAM database. It consists of two LIM domains connected by a disordered linker region. **(C)** Full length model structure of human CSRP3 modeled by a multi-template homology-based approach.

The human CSRP3 consists of two LIM domains connected by the 53-residue long disordered linker region (Figure 1). Starting from human CSRP3 (ID: NP_003467.1) as a query, a DELTA-BLAST run (Boratyn et al., 2012) was initiated against the non-redundant sequence database to yield homologs. Homologous sequences include other CSRP members (such as CSRP1 and CSRP2). Hits which are annotated as ‘hypothetical’ or ‘unnamed’ were also retained as long as they pass the thresholds on parameters. The sequence search was performed using a strict query coverage filter of 75% and a lower limit of 30% sequence identity such that only homologs that retain two LIM domains connected by a flexible linker region as query could be identified (please see Methods for details). Interestingly, 5614 homologs could be identified across a wide range of taxa, spanning 1404 species (Table 1; Supplementary Figure S1). As expected, the highest extent of homologs is observed in chordates (3211) but could be observed in lower-order organisms such as those who thrive in a freshwater habitat. Two hits were obtained from pathogenic bacterial genomes (MTV28691.1 in *Nitriliruptoraceae bacterium* ZYF776 and WP_254514402.1 in *Salmonella enterica*), which could be a contamination from hosts (please see Discussion).

**TABLE 1:** List of number of annotated and hypothetical protein IDs observed in the taxonomy at the phylum level.

S. no.	Phylum	Annotated sequences	Hypothetical sequences
1	Actinobacteria	0	1
2	Proteobacteria	0	1
3	Evosea	5	3
4	Chlorophyta	1	0
5	Streptophyta	1424	552
6	Basidiomycota	3	2
7	Zoopagomycota	1	1
8	Mucoromycota	15	11
9	Chytridiomycota	3	12
10	Porifera	2	0
11	Ctenophora	1	0
12	Cnidaria	27	0
13	Hemichordata	2	0
14	Echinodermata	14	0
15	Chordata	3021	190
16	Rotifera	10	2
17	Bryozoa	1	0
18	Annelida	1	4
19	Mollusca	45	5
20	Platyhelminthes	37	6
21	Priapulida	1	0
22	Arthropoda	148	10
23	Nematoda	32	1
24	Unclassified	12	1
	Total	**4806**	**802**

**FIGURE 2:**
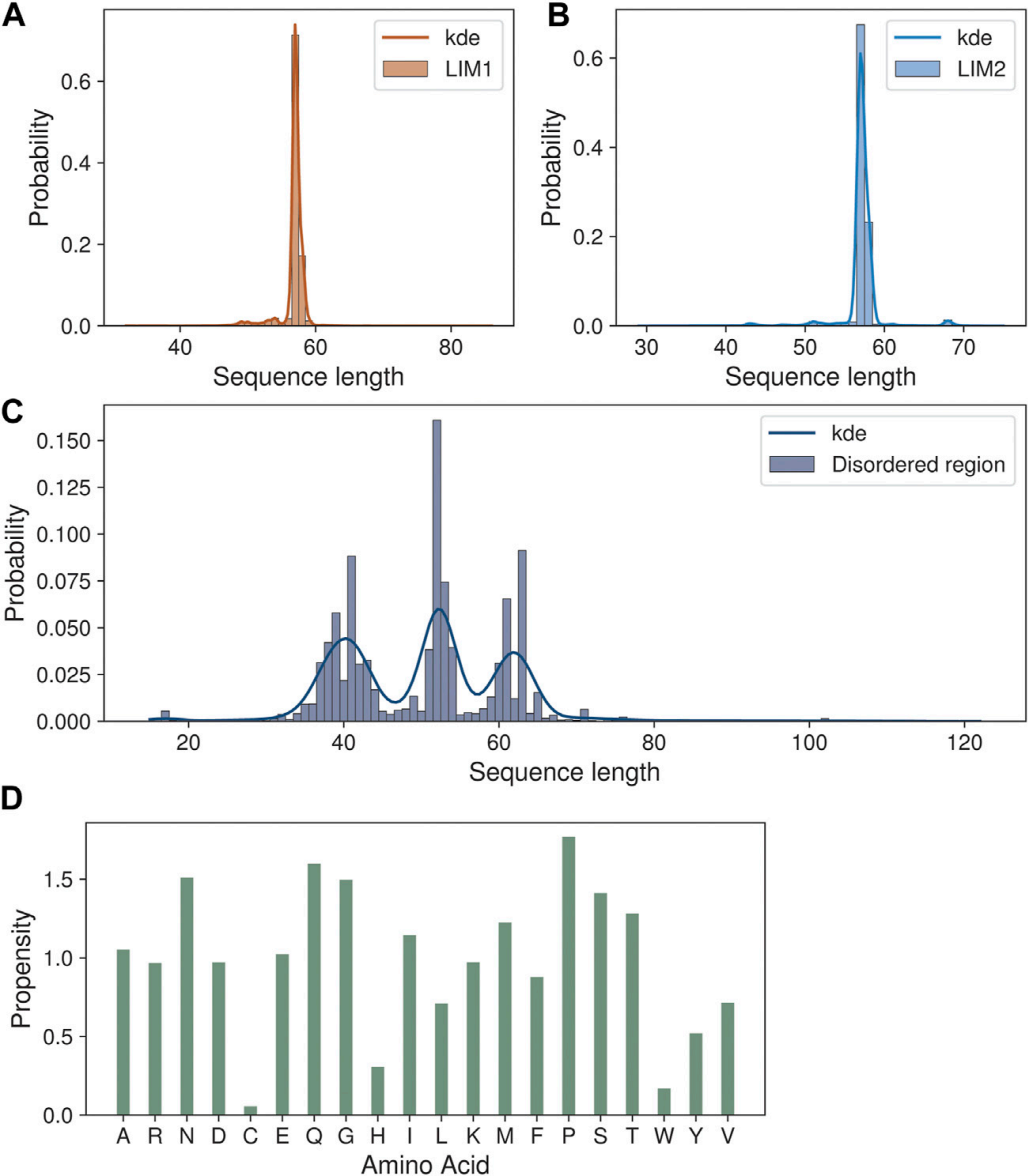
Sequence length variations in the CSRP3 homologs. **(A,B)** Histogram showing the length variation in LIM1 and LIM2 domains. **(C)** Trimodal distribution of disordered region length variation seen in the histogram plot. **(D)** Barplot highlighting the amino acid propensity in the disordered region.

**FIGURE 3:**
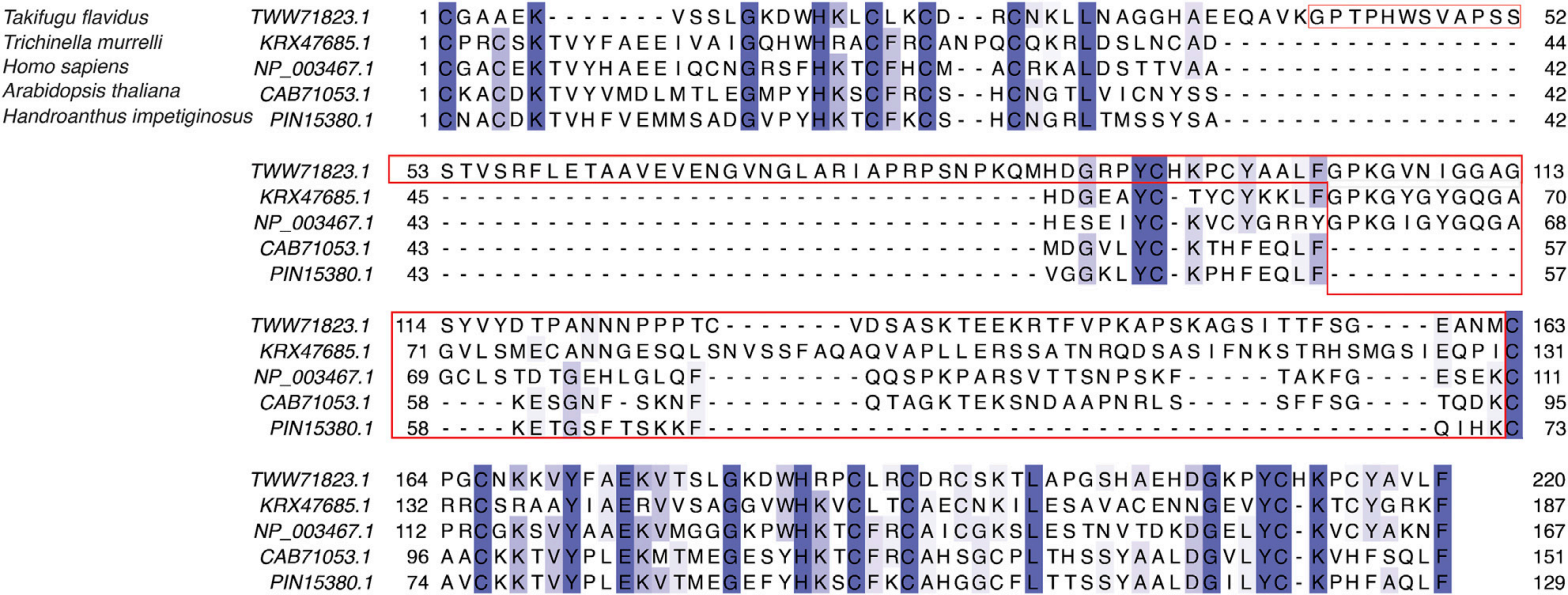
Sequence alignment of LIM domains and the disordered linker region in human CSRP3 (NP_003467.1), sansaifugu CSRP2 (TWW71823.1), nematode CSRP2 KRX47685.1, *Arabidopsis* LIM domain protein (CAB71053.1), and *Handroanthus* MLP (PIN15380.1).

**FIGURE 4:**
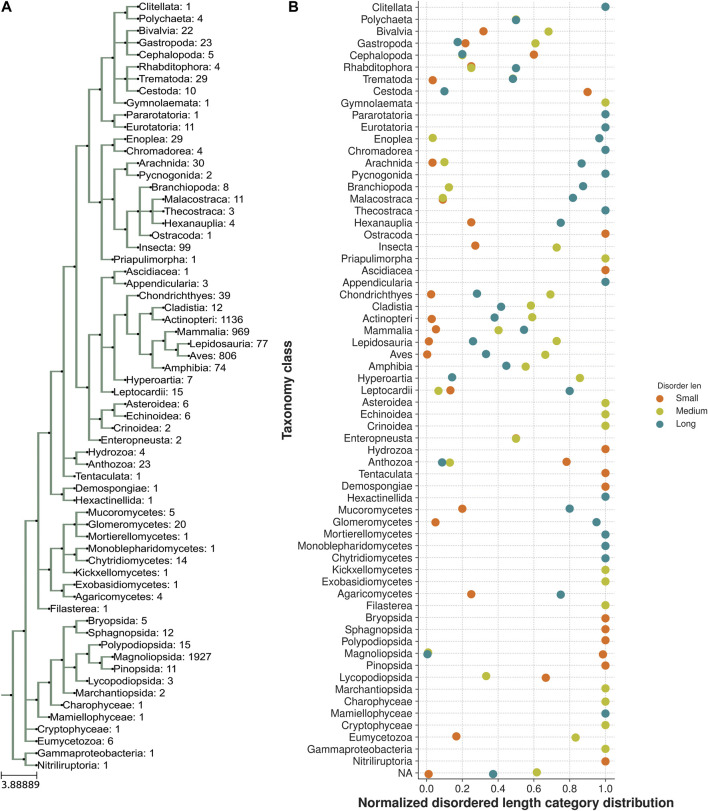
Taxonomic distribution of a trimodal disordered linker region. **(A)** Class taxa level phylogeny of CSRP3 homologs depicting the number of sequences in each class. **(B)** Normalized frequency of trimodal distribution of the linker length in each class.

We next analyzed the extent of length variations within each of the two LIM domains and the connecting flexible disordered region or linker. The distribution of the length of the LIM1 (N-terminal LIM) domain and the LIM2 (second LIM) domain was close to 57 residues, as observed in the human CSRP3. It is similar in other CRPs (CSRP1 and CSRP2) (please see Supplementary Table S2). We expected a good amount of deviation in the length of the linker region. Although the linker region is 53 amino acids long in human CSRP3, we find an interesting trimodal distribution of the linker length amongst homologs (Figure 2), with the highest peak around 52 residues. In particular, we examined those homologs which have either a longer or a shorter linker length (please see Methods for thresholds). These are in detail in Supplementary Table S3. In general, the number of homologs with unusual linker lengths is few—5 longer lengths and five shorter lengths (Supplementary Table S3). There are three CSRP sequences with longer flexible linkers from Bdelloidea (*Rotaria magna-calcarata* and *Rotaria socialis*) which thrive in freshwater. These are small microscopic animals first reported in mainland France and reproduce asexually. From our analysis, the other species where CSRP homologs retain longer-length linkers are from pufferfish and roundworm (please see Figure 3 for an alignment). From our dataset of CSRP homologs, we observe hits with appreciably shorter linkers from *Arabidopsis* and *Handroanthus impetiginosus* (pink trumpet tree; please see Figure 3 for alignment). Four others of this kind are from a variety of species—Hoatzin bird, Eurasian otter, ray-finned fish, and zebra shark. We mapped the homologs at the class level taxa and calculated the frequency of the trimodal category of a linker in each taxa. Our analysis shows that, in general, plants have a shorter linker (∼1950 homologs) while nematodes have a longer linker (29 homologs). The linker region variation at the class taxa level is highlighted in Figure 4. It is not clear how the diversity in linker lengths is likely to affect their functions. In addition, one might expect the linker region to be relatively flexible in length variation and amino acid composition.

Indeed, we calculated the propensity of 20 different amino acids to be present in the linker regions of CSRP homologs in our dataset, in comparison to their presence in the three regions, namely, LIM1 and LIM2 domains and the linker region, using standard calculations (please see Methods). We observe that amino acids such as proline, glutamine, serine, asparagine, and threonine show higher propensity at the flexible linker region. Although glycyl residues are also high in this disordered region and play an important role in sampling conformational space (please see Figure 2D), they are prevalent throughout and frequently observed within the LIM domains as well.

### 3.2 Structural analysis of human CSRP3

To understand the dynamics of the disordered linker region and LIM domains, we carried out Molecular Dynamics simulations of the full-length CSRP3 protein structure. MD simulations helps us in understanding conformation changes and interaction properties in the protein over a period of time at a given temperature and pressure. In the absence of a full-length protein structure, a homology-based model of CSRP3 protein was modeled using MODELLER 9.12 (Eswar et al., 2006). A total of 10 models were generated, and the top five DOPE scoring models were validated using SAVES 5.0 (Laskowski et al., 1993) and the ProSA server (Wiederstein and Sippl, 2007). The best model was chosen for the MD analysis for 200 ns simulation interval in triplicates using the Schrödinger Desmond suite (see Material and Methods). The same approach was followed for the *Arabidopsis* and nematode representative homolog sequences.

**FIGURE 5:**
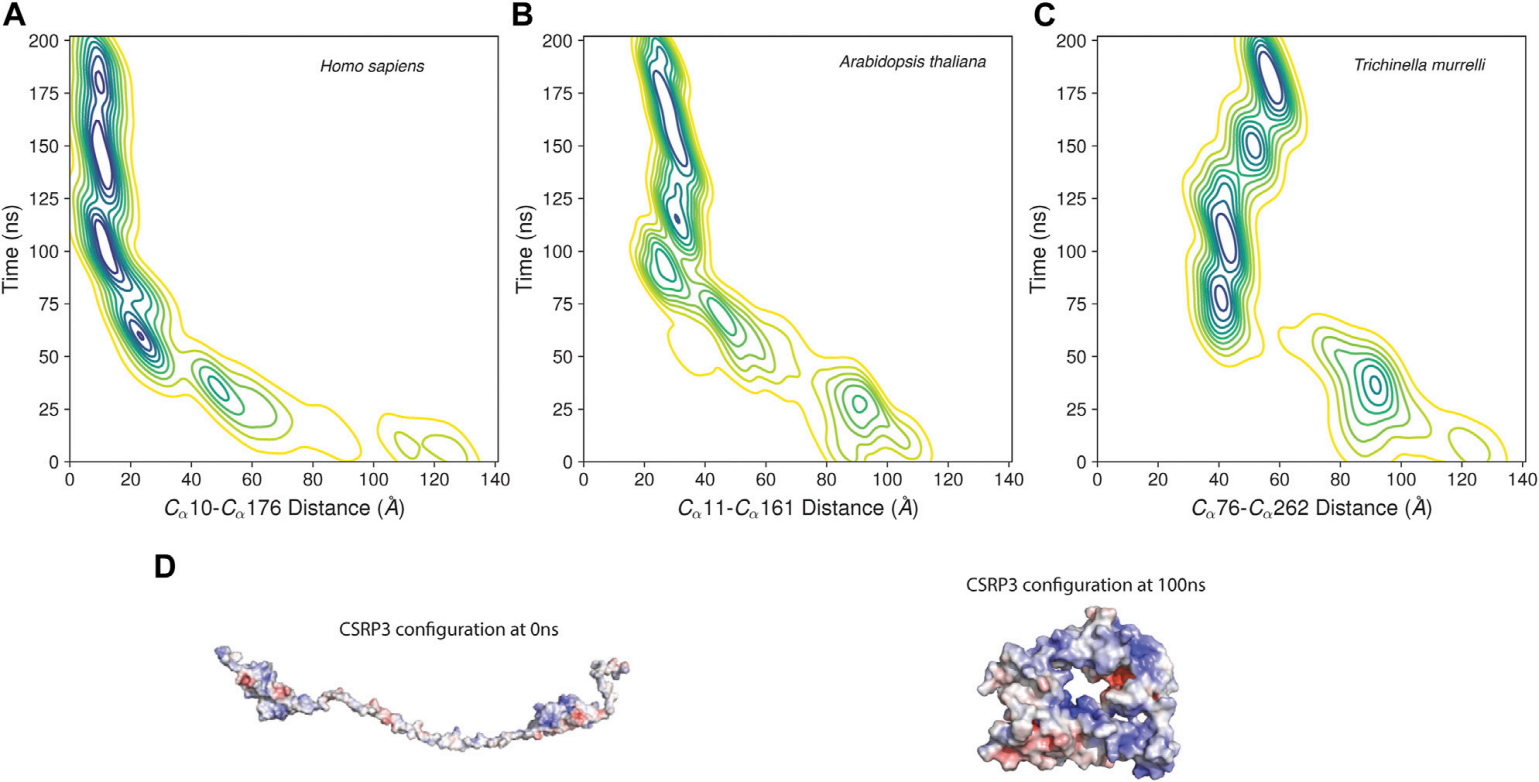
Distance between LIM1 and LIM2 as function of time in MD simulation. **(A–C)** Contour map of distance between two LIM domains of human CSRP3, *Arabidopsis* LIM domain protein, and nematode CSRP2. The distance is measured between alpha C atoms of Cys and Phe residues marking the beginning and end of N-terminal LIM and C-terminal LIM domains. **(D)** Electrostatic map of CSRP3 at the 0th frame and 100 ns frame.

The MD trajectory analysis demonstrated that there is a large structural change in CSRP3 conformation with respect to initial conformer as evident from the statistical parameter such as the root mean square distance, root mean structure fluctuation, and Radius of Gyration analyses (Supplementary Figure S2). The ROG plot shows that the structure is stabilized in ∼40 ns time interval; however, small fluctuations are observed in the RMSD plot that is eventually stabilized around 120 ns. A similar trend was observed in other two replicates (see Supplementary Figure S2 and S3). The *Arabidopsis* and nematode model structure MD simulation exhibited similar properties (Supplementary Figure S3). To further understand this behavior, we assessed the distance between two LIM domains as a function of time. The two LIM domains in human CSRP3 come together (from 120 Å to 35 Å) within ∼75 ns time and remain in close proximity throughout 200 ns simulation time (Figure 5). We carried out this LIM domain distance measurement in a shorter linker representative sequence (*Arabidopsis*) model structure and longer linker representative sequence (nematode) model structure. Although the *Arabidopsis* model structure showed a similar pattern as to human CSRP3 over a period of simulation time, nematode was a bit deviant. In this, two LIM domains seem to move away at ∼125 ns time. However, this trend is not consistent in all the replicates. The third replicate does not show movement of LIM domains once they have come together. This suggests that CSRP3 homologs with longer-length linkers might have lesser chance for the N- and C-terminal LIM domains to interact. This points to the potential role of the disordered linker region in allowing two domains to come together for a function or adapt according to interacting partners. We also investigated the secondary structural changes in the disordered region (phi–psi values). The arrow map shows that there is a dramatic change in phi–psi (φ-ψ) values from the 0th frame to 100 ns frame. The φ-ψ values were shifted from the β-sheet region to the helix region. However, there is a very small alteration from the 100 ns frame to 200 ns frame (Supplementary Figure S4). Since the previous result point to difference in φ-ψ values in the disordered region, secondary structure analysis of full-length structure was carried out in the STRIDE package (Frishman and Argos, 1995) in VMD (Humphrey et al., 1996). The analysis revealed that LIM domain secondary structures are intact in throughout the 200 ns timeline. In addition, the disordered region remained as a coil–coil structure and there is no dramatic change due to φ-ψ changes seen in the previous result (Supplementary Figure S5).

### 3.3 CSRP3 homolog functional analysis

**FIGURE 6:**
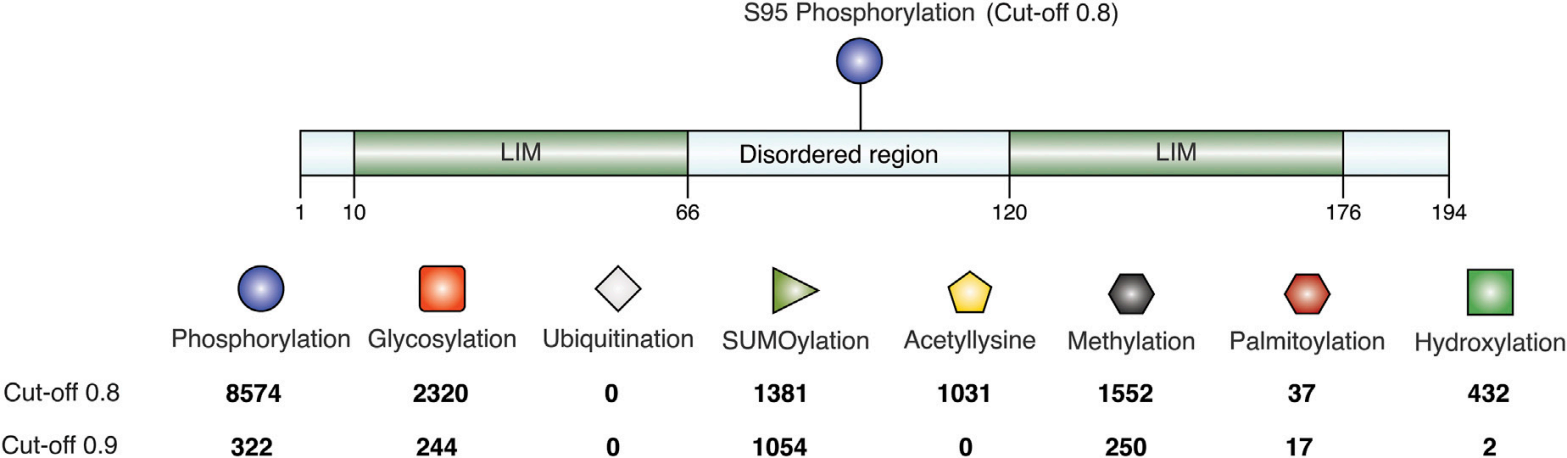
Post-translational modifications in the disordered region. The frequency is calculated at score of 0.8 and 0.9 predicted by MusiteDeep. PTMs like phosphorylation, glycosylation, ubiquitination, SUMOylation, acetylation, methylation, palmitoylation, and hydroxylation are labeled.

As seen in the previous sequence analysis results, the disordered linker region varies across CSRP3 homologs in species. Further MD analysis showed that the disordered region provides flexibility to LIM domains for dynamic adaptation. It is reported that post-translational modifications significantly change the conformer of intrinsically disordered proteins (Bah and Forman-Kay, 2016). Chiaki Nagai-Okatani and Naoto Minamino had demonstrated the functional role of O-glycosylation in the linker region of CSRP3 (Nagai-Okatani ¤a and Minamino, 2016). They highlighted that O-glycosylation plays an important role in oligomerization, and this ration is altered in a disease condition as compared to the normal condition. They postulated ∼ six glycosylation sites in the human CSRP3 linker region. We carried out the TPMs prediction in the disordered region of homologs on the MusiteDeep online server (https://www.musite.net/) (Wang et al., 2020). All the major PTMs such as phosphorylation, glycosylation, ubiquitination, SUMOylation, acetylation, methylation, palmitoylation, and hydroxylation were chosen during the prediction. Two different score cut-off values (0.8 and 0.9) were used to compare the frequency of these PTMs (Figure 6). In the human CSRP3 disordered region, only one PTM (phosphorylation of Ser 95) was predicted at the 0.8 cut-off value. Furthermore, at the cut-off value 0.9, it was absent. The O-linked_glycosylation was found to be insignificant and unreliable in human CSRP3 and needs to further investigated. In total, 8574 phosphorylation, 2320 glycosylation, 0 ubiquitination, 1381 SUMOylation, 1031 acetylation, 1552 methylation, 37 palmitoylation, and 432 hydroxylation PTMs were predicted across homologs at the score of 0.8. In contrast, 322 phosphorylation, 244 glycosylation, 0 ubiquitination, 1054 SUMOylation, 0 acetylation, 250 methylation, 17 palmitoylation, and two hydroxylation PTMs were seen at the score of 0.9. There was a significant drop in phosphorylation and glycosylation values, but the SUMOylation number remained high at the stringent value suggesting potential role of SUMOylation in the disordered region of CSRP3 homologs. The longest disordered sequence (ID = TWW71823.1 from *Takifugu flavidus*) showed similar PTMs (two phosphorylation sites) as human CSRP3 (ID = NP_003467.1) in the disordered region. Although nematode (*Trichinella murrelli*) CSRP2 (ID = KRX47685.1) was predicted to have N-linked as well as O-linked glycosylation and phosphorylation at multiple sites. Similarly, shorted disordered sequence in *Handroanthus impetiginosus* (ID = PIN15380.1) contained one methylation PTM at one site and *Arabidopsis* sequence (ID = CAB71053.1) exhibited N-linked glycosylation, hydroxylation, and phosphorylation PTMs (see Supplementary Table S4).

As reported previously, disordered sequences are advantageous for a varied range of protein–protein interactions (Fuxreiter et al., 2004; Bah and Forman-Kay, 2016), we searched a protein–protein interaction of highly varying disorder sequences KRX47685.1 (*Trichinella murrelli* CSRP2) and CAB71053.1 (*Arabidopsis* LIM domain protein) as well as human CSRP1, CSRP2, and CSRP3 protein sequences: NP_001180500.1 (human CSRP1), NP_001400464.1 (human CSRP2), and NP_003467.1 (human CSRP3). Human CSRP3 and CSRP1 show 62% sequence identity as the similar disorder region length variation. Similar observation is seen when CSRP3 is compared to CSRP2. The protein–protein interactions were searched using the sequence search option on the STRING database server (Szklarczyk et al., 2021). The similar approach was followed for the nematode sequence (ID = KRX47685.1) as well as *Arabidopsis* sequence (ID = KRX47685.1) three close homologs based on sequence similarity. Our analysis showed that even CSRP1, CSRP2, and CSRP3 have different PPIs (Supplementary Figure S6). In *Arabidopsis* and nematode close homologs, we were unable to mark similar PPIs due to annotation limitation. The full list of PPIs is provided as Supplementary Table S5.

## 4 Discussion and conclusion

CSRP proteins are important for detailed study since they are implicated in a wide variety of diseases including cardiomyopathies. Most structural characterization has been on the compact LIM domains. In this paper, we investigated the role of the disordered linker region in impacting structural and functional variations. Surprisingly, the length variations of this region show trimodal distribution, while comparing more than 5000 homologous sequences across many organisms. On the other hand, LIM domains are constrained in length variation. This suggests that there is considerable flexibility in the linker region and participates in the overall biological function of CSRP proteins. We see early evidence of CSRP3 homologs in microspecies in a freshwater habitat which do not have a circulatory system, where the precise role of CSRP is not clear. Our analysis points out that plants have a shorter linker region while nematodes seem to have a longer linker region. The mammalia taxa accommodates mixed distribution with (longer linker > medium linker >> smaller linker).

We observe that the disordered region displays conformational flexibility in a directed manner such that the two connected LIM domains could be spatially proximate. There has been limited and early evidence that LIM domains and the Zn-finger subdomains in particular facilitate dimerization of the related protein CSRP1 (Feuerstein et al., 1994). CSRP3 is present in two forms, oligomeric and monomeric (Boateng et al., 2007). These authors have proposed that the oligomeric forms are found in the cytoskeleton and monomeric forms in the nucleus. Further, the N-terminal LIM domain is implicated in dimerization/oligomerization, while the C-terminal LIM domain is involved in actin filaments (AFs) stability (Hoffmann et al., 2014). Hence, as observed by our Molecular Dynamics simulations, it is possible that the interdomain linker region may play an important role in LIM domain interaction. A longer linker tends to have relatively less domain interaction time and compactness compared to shorter and medium linker. Alternately, the open and closed conformations might influence the manner in which CSRP3 oligomerizes in the cell. The study by Papalouka et al. has previously shown that a linker region between two LIM domains of CSRP3 directly interacts with cofilin 2 (CFL2) emphasizing the role of the linker region in protein–protein interaction (Papalouka et al., 2009). Post-translational modifications are an important modulator of oligomerization. An earlier study reported that O-glycosylation influences the self-oligomerization of CSRP3 (Chiaki Nagai-Okatani and Naoto, PLoS One, 2016). Our analysis on deep learning-based prediction shows differential PTMs in CSRP3 homologs. However, O-glycosylation was not significant in the human CSRP3 linker region and more refined approach is required for finding PTMs in human CSRP3. To understand if linker region variation shows different protein–protein interactions, we also analyzed PPI for CSRP3 homologs. Human CSRPs (CSRP1, CSRP2, and CSRP3) showed shared and unique interactions (Figure 6). Other homologs exhibited different interactions that were not interpretable with limited data. Nevertheless, an extensive *in vitro* analysis is needed for a comprehensive conclusion.

## Data Availability

Publicly available datasets were analyzed in this study. This data can be found at: https://www.ncbi.nlm.nih.gov/protein/, https://www.rcsb.org/.
