# Supplementary material for: Computational analysis of the flexibility in the disordered linker region connecting LIM domains in cysteine–glycine-rich protein
Source: Front Genet. 2023 Mar 29;14:1134509. doi: 10.3389/fgene.2023.1134509 (PMC10090389; doi:10.3389/fgene.2023.1134509)
Supplement: Supplementary file 1 [file DataSheet1.zip › Supplementary_Information.pdf]

Supplementary Information

Computational Analysis of the flexibility in the disordered linker region connecting LIM domains in cysteine-glycine-rich protein (CSRP3)

Pankaj Kumar Chauhan [1] and R. Sowdhamini [1,2,3*]

[1]National Centre for Biological Sciences (Tata Institute of Fundamental Research), GKVK Campus, Bangalore Karnataka 560065 INDIA

[2]Molecular Biophysics Unit, Indian Institute of Science, Bangalore 560012 INDIA

[3]Institute of Bioinformatics and Applied Biotechnology, Bangalore, INDIA

*Correspondence: mini@ncbs.res.in

Supplementary Figures and Tables

Supplementary Figure S1: The taxonomic distribution of the CSRP3 homologs used in our analysis

This phylogeny shows the total number species of other taxonomic features of the CSRP3 homolog sequences.

Supplementary Figure S2: The statistical measures of the MD simulation runs in human CSRP3 model

(A, B, C) The Radius of Gyration (ROG), root mean square distance (RMSD) and root mean structure fluctuation (RMSF) plots for 200ns simulation time in the triplicate runs.

Supplementary Figure S3: The statistical measures of the MD simulation runs in the human CSRP3, Arabidopsis LIM domain protein and nematode CSRP2 model structures

(A) The total energy (kCal/mol) change during MD simulation time across replicates in human CSRP3. (B, C, D) The root mean square distance (RMSD) across replicates in human, Arabidopsis and nematode model structures. (E, F) The Radius of Gyration (ROG) plots in the triplicate MD runs of Arabidopsis and nematode model structures.

Supplementary Figure S4: The arrow map of variations in disordered region secondary structures (phi-psi) values in structures

The initial frame in colored in the blue while orange color depicts the final frame. The distance between initial and final values of a residue in marked by a blue color line. The glycine is marked by triangle while other residues are marked by square symbols. (A) The changes in phi-psi values of disordered region from the 0th frame to 500th frame (0ns-100ns). (B) The variations seen in phi-psi values of disordered region from the 500th frame to 1000th frame (100ns-200ns).

Supplementary Figure S5: The evolution of secondary structure elements (SSE) in MD simulation timeline

In the representation, aqua color corresponds to ‘Turn’, yellow indicates ‘Extended β-strand conformation’, white stands for ‘Coil’ (random coil), pink shows ‘alpha-helix’ and pea green depicts ‘isolated bridge’.

Supplementary Figure S6: The protein-protein interactions of candidate proteins

(A, B, C) The STRING protein-protein interactions of human CSRP1, human CSRP2, and human CSRP3 proteins.

Supplementary Table S1: List of templates used for homology modelling

The list of templates, query coverage and percent identity used in multi-template homology modelling of human CSRP3, Arabidopsis Lim domain protein and nematode CSRP2 sequences.

Supplementary Table S2: List of human CSRPs isoforms and paralogs

The list of human CSRP3 isoforms and paralogs identified in Delta-Blast search output. Partial sequences are removed. The protein-protein-interactions, tissue expression, chromosomal location, common names as well as disordered linker region sequence for each entity is also tabulated wherever possible.

Supplementary Table S3: List of CSRP homologues in the dataset with unusual length variations

The list of CSRP3 homologues with unusual length variation in LIM1, LIM2 and disordered region.

Supplementary Table S4: List of PTMs predicted in the representative proteins

List of post-translational modifications predicted in human CSRP3, Arabidopsis LIM domain protein, nematode CSRP2, sansaifugu CSRP2 and *Handroanthus* MLP.

Supplementary Table S5: List of PPIs for Arabidopsis and nematode and three homologs in each case

List PPIs for Arabidopsis LIM domain protein and its two other homologs (*Capsella rubella, Arabidopsis lyrata*), nematode CSRP2 (*Trichinella murrelli*) and its two other homologs (*Trichinella britovi, Trichinella spiralis*).
