# Supplementary material for: Computational analysis of the flexibility in the disordered linker region connecting LIM domains in cysteine–glycine-rich protein
Source: Front Genet. 2023 Mar 29;14:1134509. doi: 10.3389/fgene.2023.1134509 (PMC10090389; doi:10.3389/fgene.2023.1134509)
Supplement: Supplementary file 1 [file DataSheet1.zip › Supplementary_Tables.pdf]

Supplementary Table 1: List of templates used for homology modelling

	Query	p-value	Identity	Length	PDB
Nematode CSRP2 (KRX47685.1)	28%	2e-24	41.36%	192	1B8T_A
	17%	6e-16	53.33%	58	2O13_A
	17%	3e-10	45.00%	60	2O10_A
Arabidopsis LIM domain protein (CAB71053.1)	77%	1e-16	29.31%	192	1B8T_A
	54%	1e-09	46.55%	58	2O13_A
	54%	2e-07	41.38%	60	2O10_A
Human CSRP3 (NP_003467.1)	91%	1e-72	66.67%	192	1B8T_A
	59%	2e-36	100.00%	58	2O13_A
	60%	5e-38	100.00%	60	2O10_A

Supplementary Table 2: List of human CSRPs isoforms and orthologs

## CSRP1

PPI: GATA6, MYL9, MED14, ZYX, NUP214, FLNA, TAGLN, LMO3, MTF1, ACTN1

Tissue expression: colon, urinary bladder, seminal vesicle, prostate, fallopian tube, endometrium, smooth muscle

Isoforms/orthologs:

1. ID: NP_001180500.1
   Full name: cysteine and glycine-rich protein 1 isoform 1
   Chromosome: ch1, 1q32.1
   Protein name: CRP; CRP1; CSRP; CYRP; D1S181E; HEL-141; HEL-S-286
   Disorder region sequence:
   GPKGYGYGQGAGTLSTDKGESLGIKHEEAPGHRPTTNPNASKFAQKIGGSER
2. ID: EAW91370.1
   Full name: cysteine and glycine-rich protein 1, isoform CRA_a
   Chromosome: ch1
   Protein name:
   Disorder region sequence:
   GPKGYGYGQGAGTLSTDKGESLGIKHEEAPGHRPTTNPNASKFAQKIGGSER
3. ID: NP_001180499.1
   Full name: cysteine and glycine-rich protein 1 isoform 3
   Chromosome: ch1, 1q32.1
   Protein name: CRP; CRP1; CSRP; CYRP; D1S181E; HEL-141; HEL-S-286
   Disorder region sequence:
   GPKGYGYGQGAGTLSTDKGESLGIKHEEAPGHRPTTNPNASKFAQKIGGSER

# CSRP2 (smooth muscle)

PPI: TTLL12, TNNT2, PIAS1, TNNT3, LMO3, MYH11, TNNI3, TNNI1, TAGLN

Tissue expression: brain, retina, lung, esophagus, gallbladder, kidney, urinary bladder, prostate, vagina, fallopian tube, endometrium, placenta, cervix, breast, adipose tissue

Isoforms/orthologs:

1. ID: NP_001400469.1
   Full name: cysteine and glycine-rich protein 2 isoform c
   Chromosome: ch12, 12q21.2
   Protein name: CRP2; LMO5; SmLIM
   Disorder region sequence:
   GPKGYGYGQGAGTLNMDRGERLGIKPESVQPHRPTTNPNTSKFAQKYGGAEK
2. ID: KAI2567131.1
   Full name: cysteine and glycine rich protein 2
   Chromosome: ch12
   Protein name:
   Disorder region sequence:
   GPKGYGYGQGAGTLNMDRGERLGIKPESVQPHRPTTNPNTSKFAQKYGGAEK
3. ID: NP_001303.1
   Full name: cysteine-rich protein 2 isoform 1
   Chromosome: ch14, 14q32.33
   Protein name: CRIP; CRP2; ESP1
   Disorder region sequence:
   GPKGVNIGGAGSYIYEKPLAEGPQVTGPIEVPAARAEERKASGPPKGPSRASSVTTFTGEPNT
4. ID: NP_001257766.1
   Full name: cysteine-rich protein 2 isoform 2
   Chromosome: ch14, 14q32.33
   Protein name: CRIP; CRP2; ESP1
   Disorder region sequence:
   GPKGVNIGGAGSYIYEKPLAEGPQVTGPIEVPAARAEERKASGPPKGPSRASSVTTFTGEPNT
5. ID: NP_001400468.1
   Full name: cysteine and glycine-rich protein 2 isoform a
   Chromosome: ch12, 12q21.2
   Protein name: CRP2; LMO5; SmLIM
   Disorder region sequence:
   GPKGYGYGQGAGTLNMDRGERLGIKPESIPSCIKESCSQKQVICIYFNFTAPWRPDVMKLNGREMCCVN
   HERCFSCAIVQPHRPTTNPNTSKFAQKYGGAEK

# CSRP3 (heart muscle)

PPI: MYL2, MYOD1, MYOZ2, TCAP, ZYX, GLRX3, LDB3, MYBPC3, TTN, ACTN2

Tissue expression: heart muscle, skeletal muscle

Isoforms/orthologs:

1. ID: NP_003467.1
   Full name: cysteine and glycine-rich protein 3 isoform 1
   Chromosome: ch11, 11p15.1
   Protein name: CLP; CMD1M; CMH12; CRP3; LMO4; MLP
   Disorder region sequence:
   GPKGIGYGQGAGCLSTDTGEHLGLQFQQSPKPARSVTTSNPSKFTAKFGESEK
2. ID: EAW68356.1
   Full name: cysteine and glycine-rich protein 3
   Chromosome: ch11
   Protein name:
   Disorder region sequence:
   GPKGIGYGQGAGCLSTDTGEHLGLHSNRSPKPARSVTTSNPSKFTAKFGESEK
3. ID: NP_996805.2
   Full name: cysteine-rich protein 3 isoform 1
   Chromosome: ch 6, 6p21.1
   Protein name: CRP-3; h6LIMo; TLP; TLP-A; CRIP3

Disorder region sequence:
GPRGVNIGGVGSYLYNPPTPSPGCTTPLSPSSFSPPRPRTGLPQGKKSPPHMKTFTGETSL

4. ID: KAI4018403.1
Full name: cysteine rich protein 3
Chromosome: ch 6
Protein name:
Disorder region sequence:
GPRGVNIGGVGSYLYNPPTPSPGCTTPLSPSSFSPPRPRTGLPQGKKSPPHMKTFTGETSL

Supplementary Table 3: List of CSRP homologues in the dataset with unusual length variations

## Small LIM1 Anotn* Species Common Name Comment

CAB1335512.1 L1 < u-p *Uria aalge* *common mure bird*

CAC5376363.1 L1 < csrp *Mytilis coruscus* Mussel korea

CAG2240969.1 L1 < csrp *Mytilis edulis* blue mussel

NXV32044.1 L1 < crip2 *Rissa tridactyla* Blk[1] bird (shorter disordered region too?)

OCT58027.1 L1 < hypo *Xenopus laevis* frog (short LIM1)

XP_005679799.1 L1 <csrp2 *Capra hircus* Goat (no gaps)

XP_019912116.1 L1 <csrp3 *Esox luscious* northern pike[2]

XP_035674471.1 L1 <csrp2 *Branchiostoma floridae* lancelet fish

XP_042102481.1 L1 <csrp3 *Ovis aries* sheep

XP_035674472.1 L1 <csrp2 *Branchiostoma floridae* Lancelet fish

## Small LIM2

ABL74498.1 L2 < BLIM2b *Populus tremula* Aspen tree (disordered region gaps)

ACU17588.1 L2 < p *Glycine max* soyabean -do-

AEA39698.1 L2 < csrp1 *Epinephelus coioides* Osg[3] fish no gaps inbetween

KAF6501073.1 L2 < hypo *Molossus molossus* (vft[2] bat) C-del

NXS75146.1 L2 < csrp3 *Pandion haliaetus* (bird)

## Longer LIM1

XP_016343405.1 L1 >hypo *Sinocyclocheilus anshuiensis* ray-finned fish (between subdomains)

XP_023697041.1 L1 >csrp2 *Paramormyrops kingsleyae* elephant fish (between subdomains)

XP_047013861.1 L1 >csrp2 *Ictalurus punctatus* channel catfish N-terminal insertion (also in disordered region)

KAF4799776.1 L1 > csrp2 *Turdus rufiventris* Rb[4] thrush bird (subdomain linker)

KAF5920725.1 L1 > hypo *Diceros bicornis* black rhino (subdomain linker)

## Longer LIM2

KAF1462158.1 L2 > csrp2 *Spheniscus demersus* African penguin (disordered region also)

### Small LIM2 and Longer LIM1

MXQ87417.1 L1 > L2 <hypo *Bos mutus* wild yak (lots of gaps, longer disordered region too?)

NWJ08221.1 L1 > L2 <crip2 *Crypturellus undulates* undulated tinamou bird(longer disordered region too?)

## Longer Disordered region

TWW71823.1 D > csrp2 *Takifugu flavidus* yellowbelly pufferfish

CAF3441725.1 D > u *Rotaria socialis* Bdelloidea[5]

CAF1001371.1 D > u *Rotaria magnacalcarata* Bdelloidea[5]

XP_037835606.1 D >CSRP3 *Kryptolebias marmoratus* killifish (C-terminal overhang?)

CAF1919865.1 D > u *Rotaria magnacalcarata* Bdelloidea[5]

## Small Disordered region

XP_009937615.1 D <CSRP2 *Opisthocomus hoazin* Hoatzin bird

XP_048393952.1 D <CSRP2 *Stegostoma fasciatum* zebra shark

XP_016373137.1 D <CSRP2 *Sinocyclocheilus rhinocerous* rayfinned fish

XP_047592501.1 D <CSRP2 *Lutra lutra* Eurasian otter

PIN15380.1 D < MLP *Handroanthus impetiginosus* Pink trumpet tree

Bacterial

WP_254514402.1 hypo *Salmonella enterica*) could be host contamination

MTV28691.1 hypo *Nitriliruptoraceae bacterium*) could be host contamination

---

*Annotation in Non-Redundant database (u-p: unnamed partial; hypo: hypothetical; u: unnamed; p: partial)

1Blk: Black-legged kittiwake

2vft: velvety free-tailed

4Rb: Rufous-bellied

3Osg: Orange spotted grouper fish

5(freshwater habitat)

Alignments are provided in Figure 3 for those underlined (longest and shortest disordered regions)

Supplementary Table 4: List of PTMs predicted in the representative proteins

ID	Position	Residue	PTMscores	Cutoff=0.8
>NP_003467.1 cysteine and glycine-rich protein 3 isoform 1 [Homo sapiens]				
NP_003467.1	2	P	Hydroxyproline:0.73	None
NP_003467.1	3	K	Ubiquitination:0.661;SUMOylation:0.049;N6-acetyllysine:0.322;Methyllysine:0.77;Hydroxylysine:0.183	None
NP_003467.1	7	Y	Phosphotyrosine:0.139	None
NP_003467.1	9	Q	Pyrrolidone_carboxylic_acid:0.366	None
NP_003467.1	13	C	S-palmitoyl_cysteine:0.113	None
NP_003467.1	15	S	Phosphoserine:0.156;O-linked_glycosylation:0.254	None
NP_003467.1	16	T	Phosphothreonine:0.109;O-linked_glycosylation:0.189	None
NP_003467.1	18	T	Phosphothreonine:0.117;O-linked_glycosylation:0.31	None
NP_003467.1	25	Q	Pyrrolidone_carboxylic_acid:0.095	None
NP_003467.1	27	Q	Pyrrolidone_carboxylic_acid:0.117	None
NP_003467.1	28	Q	Pyrrolidone_carboxylic_acid:0.075	None
NP_003467.1	29	S	Phosphoserine:0.847;O-linked_glycosylation:0.339	Phosphoserine:0.847
NP_003467.1	30	P	Hydroxyproline:0.085	None
NP_003467.1	31	K	Ubiquitination:0.345;SUMOylation:0.043;N6-acetyllysine:0.151;Methyllysine:0.334;Hydroxylysine:0.035	None
NP_003467.1	32	P	Hydroxyproline:0.089	None
NP_003467.1	34	R	Methylarginine:0.117	None
NP_003467.1	35	S	Phosphoserine:0.366;O-linked_glycosylation:0.161	None
NP_003467.1	37	T	Phosphothreonine:0.225;O-linked_glycosylation:0.458	None
NP_003467.1	38	T	Phosphothreonine:0.193;O-linked_glycosylation:0.483	None
NP_003467.1	39	S	Phosphoserine:0.385;O-linked_glycosylation:0.234	None
NP_003467.1	40	N	N-linked_glycosylation:0.046	None
NP_003467.1	41	P	Hydroxyproline:0.141	None
NP_003467.1	42	S	Phosphoserine:0.517;O-linked_glycosylation:0.202	None

NP_003467.1 43 K Ubiquitination:0.505;SUMOylation:0.053;N6-acetyllysine:0.706;Methyllysine:0.224;Hydroxylysine:0.045 None

NP_003467.1 45 T Phosphothreonine:0.427;O-linked_glycosylation:0.353 None

NP_003467.1 47 K Ubiquitination:0.471;SUMOylation:0.311;N6-acetyllysine:0.641;Methyllysine:0.103;Hydroxylysine:0.025 None

NP_003467.1 51 S Phosphoserine:0.629;O-linked_glycosylation:0.25 None

NP_003467.1 53 K Ubiquitination:0.153;SUMOylation:0.068;N6-acetyllysine:0.145;Methyllysine:0.193;Hydroxylysine:0.062 None

>PIN15380.1 Regulatory protein MLP [Handroanthus impetiginosus]

PIN15380.1 1 K Ubiquitination:0.473;SUMOylation:0.062;N6-acetyllysine:0.274;Methyllysine:0.803;Hydroxylysine:0.093 Methyllysine:0.803

PIN15380.1 3 T Phosphothreonine:0.598;O-linked_glycosylation:0.443 None

PIN15380.1 5 S Phosphoserine:0.798;O-linked_glycosylation:0.208 None

PIN15380.1 7 T Phosphothreonine:0.541;O-linked_glycosylation:0.166 None

PIN15380.1 8 S Phosphoserine:0.597;O-linked_glycosylation:0.178 None

PIN15380.1 9 K Ubiquitination:0.179;SUMOylation:0.249;N6-acetyllysine:0.671;Methyllysine:0.229;Hydroxylysine:0.034 None

PIN15380.1 10 K Ubiquitination:0.242;SUMOylation:0.202;N6-acetyllysine:0.313;Methyllysine:0.291;Hydroxylysine:0.062 None

PIN15380.1 12 Q Pyrrolidone_carboxylic_acid:0.02 None

PIN15380.1 15 K Ubiquitination:0.152;SUMOylation:0.054;N6-acetyllysine:0.135;Methyllysine:0.265;Hydroxylysine:0.038 None

>CAB71053.1 LIM domain protein [Arabidopsis thaliana]

CAB71053.1 1 K Ubiquitination:0.355;SUMOylation:0.131;N6-acetyllysine:0.353;Methyllysine:0.639;Hydroxylysine:0.036 None

CAB71053.1 3 S Phosphoserine:0.684;O-linked_glycosylation:0.256 None

CAB71053.1 5 N N-linked_glycosylation:0.896 N-linked_glycosylation:0.896

CAB71053.1 7 S Phosphoserine:0.539;O-linked_glycosylation:0.104 None

CAB71053.1 8 K Ubiquitination:0.222;SUMOylation:0.206;N6-acetyllysine:0.816;Methyllysine:0.106;Hydroxylysine:0.049 N6-acetyllysine:0.816

CAB71053.1 9 N N-linked_glycosylation:0.036 None

CAB71053.1 11 Q Pyrrolidone_carboxylic_acid:0.074 None

CAB71053.1 12 T Phosphothreonine:0.211;O-linked_glycosylation:0.081 None

CAB71053.1 15 K Ubiquitination:0.203;SUMOylation:0.305;N6-acetyllysine:0.762;Methyllysine:0.043;Hydroxylysine:0.035 None

CAB71053.1 16 T Phosphothreonine:0.138;O-linked_glycosylation:0.069 None

CAB71053.1 18 K Ubiquitination:0.234;SUMOylation:0.05;N6-acetyllysine:0.398;Methyllysine:0.077;Hydroxylysine:0.04 None

CAB71053.1 19 S Phosphoserine:0.44;O-linked_glycosylation:0.059 None

CAB71053.1 20 N N-linked_glycosylation:0.032 None

CAB71053.1 24 P Hydroxyproline:0.103 None

CAB71053.1 25 N N-linked_glycosylation:0.037 None

CAB71053.1 26 R Methylarginine:0.189 None

CAB71053.1 28 S Phosphoserine:0.69;O-linked_glycosylation:0.155 None

CAB71053.1	29	S	Phosphoserine:0.847;O-linked_glycosylation:0.15	Phosphoserine:0.847

CAB71053.1	32	S	Phosphoserine:0.652;O-linked_glycosylation:0.211	None

CAB71053.1	34	T	Phosphothreonine:0.723;O-linked_glycosylation:0.11	None

CAB71053.1	35	Q	Pyrrolidone_carboxylic_acid:0.024	None

CAB71053.1	37	K	Ubiquitination:0.227;SUMOylation:0.064;N6-acetyllysine:0.16;Methyllysine:0.248;Hydroxylysine:0.063	None

>KRX47685.1 Cysteine and glycine-rich protein 2 [Trichinella murrelli]

KRX47685.1	2	P	Hydroxyproline:0.719	None

KRX47685.1	3	K	Ubiquitination:0.588;SUMOylation:0.047;N6-acetyllysine:0.236;Methyllysine:0.765;Hydroxylysine:0.232	None

KRX47685.1	5	Y	Phosphotyrosine:0.09	None

KRX47685.1	7	Y	Phosphotyrosine:0.107	None

KRX47685.1	9	Q	Pyrrolidone_carboxylic_acid:0.264	None

KRX47685.1	15	S	Phosphoserine:0.228;O-linked_glycosylation:0.232	None

KRX47685.1	18	C	S-palmitoyl_cysteine:0.037	None

KRX47685.1	20	N	N-linked_glycosylation:0.032	None

KRX47685.1	21	N	N-linked_glycosylation:0.034	None

KRX47685.1	24	S	Phosphoserine:0.084;O-linked_glycosylation:0.081	None

KRX47685.1	25	Q	Pyrrolidone_carboxylic_acid:0.035	None

KRX47685.1	27	S	Phosphoserine:0.108;O-linked_glycosylation:0.079	None

KRX47685.1	28	N	N-linked_glycosylation:0.899	N-linked_glycosylation:0.899

KRX47685.1	30	S	Phosphoserine:0.103;O-linked_glycosylation:0.069	None

KRX47685.1	31	S	Phosphoserine:0.169;O-linked_glycosylation:0.065	None

KRX47685.1	34	Q	Pyrrolidone_carboxylic_acid:0.062	None

KRX47685.1	36	Q	Pyrrolidone_carboxylic_acid:0.097	None

KRX47685.1	39	P	Hydroxyproline:0.067	None

KRX47685.1	43	R	Methylarginine:0.181	None

KRX47685.1	44	S	Phosphoserine:0.228;O-linked_glycosylation:0.102	None

KRX47685.1	45	S	Phosphoserine:0.232;O-linked_glycosylation:0.145	None

KRX47685.1	47	T	Phosphothreonine:0.17;O-linked_glycosylation:0.104	None

KRX47685.1	48	N	N-linked_glycosylation:0.033	None

KRX47685.1	49	R	Methylarginine:0.366	None

KRX47685.1	50	Q	Pyrrolidone_carboxylic_acid:0.638	None

KRX47685.1	52	S	Phosphoserine:0.853;O-linked_glycosylation:0.092	Phosphoserine:0.853

KRX47685.1	54	S	Phosphoserine:0.675;O-linked_glycosylation:0.146	None

KRX47685.1	57	N	N-linked_glycosylation:0.817	N-linked_glycosylation:0.817

KRX47685.1	58	K	Ubiquitination:0.384;SUMOylation:0.068;N6-acetyllysine:0.751;Methyllysine:0.318;Hydroxylysine:0.049	None

KRX47685.1	59	S	Phosphoserine:0.551;O-linked_glycosylation:0.122	None

KRX47685.1	60	T	Phosphothreonine:0.246;O-linked_glycosylation:0.142	None

KRX47685.1	61	R	Methylarginine:0.216	None

KRX47685.1	63	S	Phosphoserine:0.853;O-linked_glycosylation:0.124	Phosphoserine:0.853

KRX47685.1	66	S	Phosphoserine:0.871;O-linked_glycosylation:0.197	Phosphoserine:0.871

KRX47685.1	69	Q	Pyrrolidone_carboxylic_acid:0.019	None

KRX47685.1	70	P	Hydroxyproline:0.168	None

>TWW71823.1 Cysteine-rich protein 2 [Takifugu flavidus]

TWW71823.1	2	P	Hydroxyproline:0.727	None

TWW71823.1	3	T	Phosphothreonine:0.613;O-linked_glycosylation:0.664	None

TWW71823.1	4	P	Hydroxyproline:0.635	None

TWW71823.1	7	S	Phosphoserine:0.388;O-linked_glycosylation:0.481	None

TWW71823.1	10	P	Hydroxyproline:0.3	None

TWW71823.1	11	S	Phosphoserine:0.262;O-linked_glycosylation:0.373	None

TWW71823.1	12	S	Phosphoserine:0.238;O-linked_glycosylation:0.276	None

TWW71823.1	13	S	Phosphoserine:0.335;O-linked_glycosylation:0.122	None

TWW71823.1	14	T	Phosphothreonine:0.181;O-linked_glycosylation:0.137	None

TWW71823.1	16	S	Phosphoserine:0.156;O-linked_glycosylation:0.086	None

TWW71823.1	17	R	Methylarginine:0.061	None

TWW71823.1	21	T	Phosphothreonine:0.109;O-linked_glycosylation:0.084	None

TWW71823.1	28	N	N-linked_glycosylation:0.033	None

TWW71823.1	31	N	N-linked_glycosylation:0.035	None

TWW71823.1	35	R	Methylarginine:0.11	None

TWW71823.1	38	P	Hydroxyproline:0.418	None

TWW71823.1	39	R	Methylarginine:0.076	None

TWW71823.1	40	P	Hydroxyproline:0.739	None

TWW71823.1	41	S	Phosphoserine:0.299;O-linked_glycosylation:0.246	None

TWW71823.1	42	N	N-linked_glycosylation:0.035	None

TWW71823.1	43	P	Hydroxyproline:0.723	None

TWW71823.1	44	K	Ubiquitination:0.506;SUMOylation:0.037;N6-acetyllysine:0.229;Methyllysine:0.577;Hydroxylysine:0.208	None

TWW71823.1	45	Q	Pyrrolidone_carboxylic_acid:0.209	None

TWW71823.1	50	R	Methylarginine:0.162	None

TWW71823.1	51	P	Hydroxyproline:0.41	None

TWW71823.1	52	Y	Phosphotyrosine:0.087	None

TWW71823.1	53	C	S-palmitoyl_cysteine:0.058	None

TWW71823.1	55	K	Ubiquitination:0.331;SUMOylation:0.034;N6-acetyllysine:0.235;Methyllysine:0.153;Hydroxylysine:0.332	None

TWW71823.1	56	P	Hydroxyproline:0.52	None

TWW71823.1	57	C	S-palmitoyl_cysteine:0.059	None

TWW71823.1	58	Y	Phosphotyrosine:0.109	None
TWW71823.1	64	P	Hydroxyproline:0.2	None
TWW71823.1	65	K	Ubiquitination:0.361;SUMOylation:0.061;N6-acetyllysine:0.153;Methyllysine:0.11;Hydroxylysine:0.466	None
TWW71823.1	68	N	N-linked_glycosylation:0.032	None
TWW71823.1	74	S	Phosphoserine:0.14;O-linked_glycosylation:0.159	None
TWW71823.1	75	Y	Phosphotyrosine:0.19	None
TWW71823.1	77	Y	Phosphotyrosine:0.75	None
TWW71823.1	79	T	Phosphothreonine:0.433;O-linked_glycosylation:0.403	None
TWW71823.1	80	P	Hydroxyproline:0.118	None
TWW71823.1	82	N	N-linked_glycosylation:0.036	None
TWW71823.1	83	N	N-linked_glycosylation:0.035	None
TWW71823.1	84	N	N-linked_glycosylation:0.035	None
TWW71823.1	85	P	Hydroxyproline:0.252	None
TWW71823.1	86	P	Hydroxyproline:0.25	None
TWW71823.1	87	P	Hydroxyproline:0.196	None
TWW71823.1	88	T	Phosphothreonine:0.138;O-linked_glycosylation:0.679	None
TWW71823.1	89	C	S-palmitoyl_cysteine:0.144	None
TWW71823.1	92	S	Phosphoserine:0.249;O-linked_glycosylation:0.418	None
TWW71823.1	94	S	Phosphoserine:0.162;O-linked_glycosylation:0.591	None
TWW71823.1	95	K	Ubiquitination:0.237;SUMOylation:0.12;N6-acetyllysine:0.217;Methyllysine:0.18;Hydroxylysine:0.035	None
TWW71823.1	96	T	Phosphothreonine:0.107;O-linked_glycosylation:0.351	None
TWW71823.1	99	K	Ubiquitination:0.224;SUMOylation:0.048;N6-acetyllysine:0.183;Methyllysine:0.095;Hydroxylysine:0.021	None
TWW71823.1	100	R	Methylarginine:0.029	None
TWW71823.1	101	T	Phosphothreonine:0.248;O-linked_glycosylation:0.322	None
TWW71823.1	104	P	Hydroxyproline:0.168	None
TWW71823.1	105	K	Ubiquitination:0.484;SUMOylation:0.052;N6-acetyllysine:0.466;Methyllysine:0.092;Hydroxylysine:0.063	None
TWW71823.1	107	P	Hydroxyproline:0.203	None
TWW71823.1	108	S	Phosphoserine:0.759;O-linked_glycosylation:0.143	None
TWW71823.1	109	K	Ubiquitination:0.664;SUMOylation:0.062;N6-acetyllysine:0.271;Methyllysine:0.155;Hydroxylysine:0.051	None
TWW71823.1	112	S	Phosphoserine:0.872;O-linked_glycosylation:0.128	Phosphoserine:0.872
TWW71823.1	114	T	Phosphothreonine:0.485;O-linked_glycosylation:0.212	None
TWW71823.1	115	T	Phosphothreonine:0.616;O-linked_glycosylation:0.179	None
TWW71823.1	117	S	Phosphoserine:0.802;O-linked_glycosylation:0.196	Phosphoserine:0.802
TWW71823.1	121	N	N-linked_glycosylation:0.046	None

Supplementary Table 5: List of PPIs for Arabidopsis and nematode and three homologues in each case

PPI in Arabidopsis thaliana and homologues
Species: Arabidopsis thaliana
GeneID: CAB71053.1

#node1	node2	node1_string_id	node2_string_id	neighborhood_on_chromosome	gene_fusion	phylogenetic_cooccurrence	homology	coexpression	experimentally_determined_interaction	database_annotated	automated_textmining	combined_score
ACT12	CPB	3702.AT3G46520.1	3702.AT1G71790.1	0	0	0	0	0.050	0.747	0.638	0.679	0.968
ACT12	VLN1	3702.AT3G46520.1	3702.AT2G29890.3	0	0	0	0	0.046	0.575	0.612	0.717	0.949
ACT12	PLIM2c	3702.AT3G46520.1	3702.AT3G61230.1	0	0	0	0	0.045	0.185	0.260	0.480	0.660
ACT12	FIM5	3702.AT3G46520.1	3702.AT5G35700.1	0	0	0	0	0.045	0.468	0.260	0.710	0.876
ACT12	VLN4	3702.AT3G46520.1	3702.AT4G30160.2	0	0	0	0	0.046	0.184	0.612	0.686	0.892
ACT12	VLN5	3702.AT3G46520.1	3702.AT5G57320.1	0	0	0	0	0.046	0.184	0.612	0.689	0.893
AT1G01770	SDR1	3702.AT1G01770.1	3702.AT3G61220.2	0	0	0	0	0	0	0	0.660	0.660
AT1G01770	PLIM2c	3702.AT1G01770.1	3702.AT3G61230.1	0	0	0	0	0	0	0	0.806	0.806
AT3G28630	VLN1	3702.AT3G28630.1	3702.AT2G29890.3	0	0	0	0	0	0	0	0.449	0.449
AT3G28630	VLN4	3702.AT3G28630.1	3702.AT4G30160.2	0	0	0	0	0	0	0	0.482	0.482
AT3G28630	VLN5	3702.AT3G28630.1	3702.AT5G57320.1	0	0	0	0	0	0	0	0.638	0.638
AT3G28630	PLIM2c	3702.AT3G28630.1	3702.AT3G61230.1	0	0	0	0	0	0	0	0.871	0.871
AT3G28630	FIM5	3702.AT3G28630.1	3702.AT5G35700.1	0	0	0	0	0	0	0	0.874	0.874
AT5G05650	PLIM2c	3702.AT5G05650.1	3702.AT3G61230.1	0	0	0	0	0.058	0	0	0.635	0.641
CPB	VLN4	3702.AT1G71790.1	3702.AT4G30160.2	0	0	0	0	0	0	0	0.556	0.556
CPB	VLN5	3702.AT1G71790.1	3702.AT5G57320.1	0	0	0	0	0	0	0	0.556	0.556
CPB	VLN1	3702.AT1G71790.1	3702.AT2G29890.3	0	0	0	0	0	0	0	0.556	0.556
CPB	PLIM2c	3702.AT1G71790.1	3702.AT3G61230.1	0	0	0	0	0	0.524	0.448	0.326	0.807
CPB	FIM5	3702.AT1G71790.1	3702.AT5G35700.1	0	0	0	0	0.044	0.858	0.448	0.616	0.967
FIM5	VLN1	3702.AT5G35700.1	3702.AT2G29890.3	0	0	0	0	0	0.079	0	0.859	0.865
FIM5	PLIM2c	3702.AT5G35700.1	3702.AT3G61230.1	0	0	0	0	0	0	0	0.724	0.724
FIM5	VLN5	3702.AT5G35700.1	3702.AT5G57320.1	0	0	0	0	0	0.079	0	0.886	0.891
PLIM2c	VLN1	3702.AT3G61230.1	3702.AT2G29890.3	0	0	0	0	0.045	0.102	0	0.621	0.647
PLIM2c	SDR1	3702.AT3G61230.1	3702.AT3G61220.2	0	0	0	0	0	0	0	0.634	0.634
PLIM2c	VLN5	3702.AT3G61230.1	3702.AT5G57320.1	0	0	0	0	0.045	0.102	0	0.643	0.667
PLIM2c	VLN4	3702.AT3G61230.1	3702.AT4G30160.2	0	0	0	0	0.045	0.102	0	0.785	0.800

Species: Capsella rubella
GeneID: XP_006292212.1

#node1 node2 node1_string_id node2_string_id neighborhood_on_chromosome
gene_fusion phylogenetic_cooccurrence homology coexpression
experimentally_determined_interaction database_annotated
automated_textmining combined_score
XP_006279546.1 XP_006279963.1 81985.XP_006279546.1
81985.XP_006279963.1 0 0 0 0 0 0 0
0.609 0.609
XP_006279546.1 XP_006292212.1 81985.XP_006279546.1
81985.XP_006292212.1 0 0 0 0 0.048 0.229 0.578
0.219 0.725
XP_006279963.1 XP_006307234.1 81985.XP_006279963.1
81985.XP_006307234.1 0 0 0 0 0 0 0
0.451 0.451
XP_006279963.1 XP_006280671.1 81985.XP_006279963.1
81985.XP_006280671.1 0 0 0 0 0 0 0
0.609 0.609
XP_006279963.1 XP_006280524.1 81985.XP_006279963.1
81985.XP_006280524.1 0 0 0 0 0 0 0
0.609 0.609
XP_006279963.1 XP_006283450.1 81985.XP_006279963.1
81985.XP_006283450.1 0 0 0 0 0 0 0
0.641 0.641
XP_006279963.1 XP_006293994.1 81985.XP_006279963.1
81985.XP_006293994.1 0 0 0 0 0 0 0
0.671 0.671
XP_006279963.1 XP_006292212.1 81985.XP_006279963.1
81985.XP_006292212.1 0 0 0 0 0 0 0
0.694 0.694
XP_006280524.1 XP_006292212.1 81985.XP_006280524.1
81985.XP_006292212.1 0 0 0 0 0.048 0.229 0.578
0.219 0.725
XP_006280671.1 XP_006292212.1 81985.XP_006280671.1
81985.XP_006292212.1 0 0 0 0 0.048 0.229 0.578
0.219 0.725
XP_006282593.1 XP_006292212.1 81985.XP_006282593.1
81985.XP_006292212.1 0 0 0 0 0 0.105 0.663
0.045 0.686
XP_006282593.1 XP_006283638.1 81985.XP_006282593.1
81985.XP_006283638.1 0 0 0.480 0.976 0 0 0.800 0
0.802
XP_006283450.1 XP_006292212.1 81985.XP_006283450.1
81985.XP_006292212.1 0 0 0 0 0.048 0.229 0.578
0.509 0.827
XP_006283638.1 XP_006292212.1 81985.XP_006283638.1
81985.XP_006292212.1 0 0 0 0 0 0.105 0.663
0.045 0.686
XP_006292212.1 XP_006307234.1 81985.XP_006292212.1
81985.XP_006307234.1 0 0 0 0 0.048 0.229 0.578
0.219 0.725
XP_006292212.1 XP_006302717.1 81985.XP_006292212.1
81985.XP_006302717.1 0 0 0 0 0 0.452 0.476
0.312 0.785
XP_006292212.1 XP_006293994.1 81985.XP_006292212.1
81985.XP_006293994.1 0 0 0 0 0.048 0.229 0.578
0.550 0.841

Species: Arabidopsis lyrata
GeneID: 020881577.1
#node1 node2 node1_string_id node2_string_id neighborhood_on_chromosome
gene_fusion phylogenetic_cooccurrence homology coexpression
experimentally_determined_interaction database_annotated
automated_textmining combined_score
Al_scaffold_0008_3264 fgenesh1_pm.C_scaffold_8000996
59689.Al_scaffold_0008_3264 59689.fgenesh1_pm.C_scaffold_8000996 0
0 0 0 0 0 0 0.590 0.590
Al_scaffold_0008_3264 fgenesh2_kg.5__2591__AT3G61230.1
59689.Al_scaffold_0008_3264 59689.fgenesh2_kg.5__2591__AT3G61230.1 0
0 0 0 0.049 0.225 0.620 0.254 0.763

fgenesh1_pg.C_scaffold_8002683 fgenesh1_pm.C_scaffold_8000996
59689.fgenesh1_pg.C_scaffold_8002683 59689.fgenesh1_pm.C_scaffold_8000996
0 0 0 0 0 0 0 0.590 0.590
fgenesh1_pg.C_scaffold_8002683 fgenesh2_kg.5__2591__AT3G61230.1
59689.fgenesh1_pg.C_scaffold_8002683
59689.fgenesh2_kg.5__2591__AT3G61230.1 0 0 0 0 0.049
0.225 0.620 0.254 0.763
fgenesh1_pm.C_scaffold_4001729 fgenesh1_pm.C_scaffold_8000996
59689.fgenesh1_pm.C_scaffold_4001729 59689.fgenesh1_pm.C_scaffold_8000996
0 0 0 0 0 0 0 0.658 0.658
fgenesh1_pm.C_scaffold_4001729 fgenesh2_kg.5__2591__AT3G61230.1
59689.fgenesh1_pm.C_scaffold_4001729
59689.fgenesh2_kg.5__2591__AT3G61230.1 0 0 0 0 0.049
0.225 0.620 0.577 0.865
fgenesh1_pm.C_scaffold_7000309 fgenesh1_pm.C_scaffold_8000996
59689.fgenesh1_pm.C_scaffold_7000309 59689.fgenesh1_pm.C_scaffold_8000996
0 0 0 0 0 0 0 0.624 0.624
fgenesh1_pm.C_scaffold_7000309 fgenesh2_kg.5__2591__AT3G61230.1
59689.fgenesh1_pm.C_scaffold_7000309
59689.fgenesh2_kg.5__2591__AT3G61230.1 0 0 0 0 0.049
0.225 0.620 0.542 0.854
fgenesh1_pm.C_scaffold_8000996 scaffold_103905.1
59689.fgenesh1_pm.C_scaffold_8000996 59689.scaffold_103905.1 0 0
0 0 0 0 0 0.449 0.449
fgenesh1_pm.C_scaffold_8000996 fgenesh2_kg.1__2103__AT1G19270.1
59689.fgenesh1_pm.C_scaffold_8000996
59689.fgenesh2_kg.1__2103__AT1G19270.1 0 0 0 0 0 0
0 0.449 0.449
fgenesh1_pm.C_scaffold_8000996 fgenesh2_kg.8__2755__AT5G66640.1
59689.fgenesh1_pm.C_scaffold_8000996
59689.fgenesh2_kg.8__2755__AT5G66640.1 0 0 0 0 0 0
0 0.590 0.590
fgenesh1_pm.C_scaffold_8000996 fgenesh2_kg.8__2758__AT5G66610.1
59689.fgenesh1_pm.C_scaffold_8000996
59689.fgenesh2_kg.8__2758__AT5G66610.1 0 0 0 0 0 0
0 0.590 0.590
fgenesh1_pm.C_scaffold_8000996 fgenesh2_kg.5__2591__AT3G61230.1
59689.fgenesh1_pm.C_scaffold_8000996
59689.fgenesh2_kg.5__2591__AT3G61230.1 0 0 0 0 0 0
0 0.698 0.698
fgenesh2_kg.1__2103__AT1G19270.1 fgenesh2_kg.5__2591__AT3G61230.1
59689.fgenesh2_kg.1__2103__AT1G19270.1
59689.fgenesh2_kg.5__2591__AT3G61230.1 0 0 0 0 0.049
0.225 0.620 0.254 0.763
fgenesh2_kg.2__1430__AT1G71790.1 fgenesh2_kg.5__2591__AT3G61230.1
59689.fgenesh2_kg.2__1430__AT1G71790.1
59689.fgenesh2_kg.5__2591__AT3G61230.1 0 0 0 0 0
0.674 0.476 0.337 0.876
fgenesh2_kg.5__2591__AT3G61230.1 scaffold_103905.1
59689.fgenesh2_kg.5__2591__AT3G61230.1 59689.scaffold_103905.1 0 0
0 0 0.049 0.225 0.620 0.254 0.763
fgenesh2_kg.5__2591__AT3G61230.1 fgenesh2_kg.8__2755__AT5G66640.1
59689.fgenesh2_kg.5__2591__AT3G61230.1
59689.fgenesh2_kg.8__2755__AT5G66640.1 0 0 0 0 0.049
0.225 0.620 0.254 0.763
fgenesh2_kg.5__2591__AT3G61230.1 fgenesh2_kg.8__2758__AT5G66610.1
59689.fgenesh2_kg.5__2591__AT3G61230.1
59689.fgenesh2_kg.8__2758__AT5G66610.1 0 0 0 0 0.049
0.225 0.620 0.254 0.763

PPI in Trichinella murrelli and homologues
_
Species: Trichinella murrelli
GeneID: KRX47685.1
#node1 node2 node1_string_id node2_string_id neighborhood_on_chromosome
gene_fusion phylogenetic_cooccurrence homology coexpression

experimentally_determined_interaction database_annotated
automated_textmining combined_score
Actn tni-3 144512.A0A0V0TZT7 144512.A0A0V0TDJ3 0 0 0 0
0.606 0.641 0.666 0.642 0.981
Actn CSRP2 144512.A0A0V0TZT7 144512.A0A0V0U995 0 0 0 0
0.321 0.328 0 0.212 0.609
CSRP2 tni-3 144512.A0A0V0U995 144512.A0A0V0TDJ3 0 0 0 0
0.326 0 0 0.207 0.443
CSRP2 TRIP4 144512.A0A0V0U995 144512.A0A0V0UFL2 0 0 0 0 0
0.248 0 0.253 0.414

Species: Trichinella britovi
GeneID: KRY58785.1
#node1 node2 node1_string_id node2_string_id neighborhood_on_chromosome
gene_fusion phylogenetic_cooccurrence homology coexpression
experimentally_determined_interaction database_annotated
automated_textmining combined_score
A0A0V1CIG5 tni-3 45882.A0A0V1CIG5 45882.A0A0V1CEQ8 0 0 0 0
0.417 0.407 0.876 0.681 0.984
A0A0V1CIG5 Pxn 45882.A0A0V1CIG5 45882.A0A0V1CM28 0 0 0 0
0.394 0 0.636 0.266 0.824
A0A0V1CIG5 tnc-2 45882.A0A0V1CIG5 45882.A0A0V1CQW1 0 0 0 0
0.415 0 0 0.605 0.759
A0A0V1CIG5 Actn 45882.A0A0V1CIG5 45882.A0A0V1CWE6 0 0 0 0
0.632 0.811 0.626 0.700 0.991
A0A0V1CIG5 CSRP2 45882.A0A0V1CIG5 45882.A0A0V1DBE3 0 0 0 0
0.492 0 0 0.313 0.636
A0A0V1D4E7 CSRP2 45882.A0A0V1D4E7 45882.A0A0V1DBE3 0 0 0 0
0 0.588 0 0 0.588
Actn tni-3 45882.A0A0V1CWE6 45882.A0A0V1CEQ8 0 0 0 0
0.606 0.641 0.666 0.642 0.981
Actn unc-97 45882.A0A0V1CWE6 45882.A0A0V1CII0 0 0 0 0
0.133 0 0.581 0.699 0.881
Actn Pxn 45882.A0A0V1CWE6 45882.A0A0V1CM28 0 0 0 0
0.250 0 0.607 0.782 0.930
Actn tnc-2 45882.A0A0V1CWE6 45882.A0A0V1CQW1 0 0 0 0.305
0.500 0 0 0.469 0.655
Actn CSRP2 45882.A0A0V1CWE6 45882.A0A0V1DBE3 0 0 0 0
0.677 0.590 0 0.440 0.919
Ascc1 TRIP4 45882.A0A0V1D8B5 45882.A0A0V1DAI0 0 0 0 0
0.145 0.795 0 0.701 0.943
Ascc1 CSRP2 45882.A0A0V1D8B5 45882.A0A0V1DBE3 0 0 0 0 0
0.306 0 0.457 0.607
CSRP2 tni-3 45882.A0A0V1DBE3 45882.A0A0V1CEQ8 0 0 0 0
0.698 0 0 0.378 0.804
CSRP2 unc-97 45882.A0A0V1DBE3 45882.A0A0V1CII0 0 0 0 0.152
0.141 0.203 0 0.499 0.575
CSRP2 Pxn 45882.A0A0V1DBE3 45882.A0A0V1CM28 0 0 0 0.187
0.416 0 0 0.664 0.724
CSRP2 tnc-2 45882.A0A0V1DBE3 45882.A0A0V1CQW1 0 0 0 0
0.411 0 0 0.328 0.587
CSRP2 RAB10 45882.A0A0V1DBE3 45882.A0A0V1CYH0 0 0 0 0 0
0.568 0 0.142 0.613
CSRP2 TRIP4 45882.A0A0V1DBE3 45882.A0A0V1DAI0 0 0 0 0 0
0.306 0 0.542 0.669
Pxn unc-97 45882.A0A0V1CM28 45882.A0A0V1CII0 0 0 0 0.382
0.144 0.305 0.636 0.800 0.880
tnc-2 tni-3 45882.A0A0V1CQW1 45882.A0A0V1CEQ8 0 0 0 0
0.466 0.817 0 0.699 0.968

Species: Trichinella spiralis
GeneID: KRY35008.1
#node1 node2 node1_string_id node2_string_id neighborhood_on_chromosome
gene_fusion phylogenetic_cooccurrence homology coexpression
experimentally_determined_interaction database_annotated
automated_textmining combined_score
CSRP2 EFV47728 6334.EFV57870 6334.EFV47728 0 0 0 0 0.171
0.133 0.331 0.273 0.603

CSRP2 EFV48665 6334.EFV57870 6334.EFV48665 0 0 0 0 0.171
0.133 0.331 0.273 0.603
CSRP2 LPP 6334.EFV57870 6334.EFV52047 0 0 0 0 0.090 0.090
0.159 0.495 0.601
CSRP2 unc-15 6334.EFV57870 6334.EFV52403 0 0 0 0 0.608 0.096
0.127 0.048 0.666
CSRP2 EFV55561 6334.EFV57870 6334.EFV55561 0 0 0 0 0.171
0.133 0.331 0.273 0.603
CSRP2 mup-2 6334.EFV57870 6334.EFV56156 0 0 0 0 0.584 0.044
0.129 0.127 0.657
CSRP2 SMTNL2 6334.EFV57870 6334.EFV61840 0 0 0 0 0.163 0
0.510 0 0.572
CSRP2 EFV60001 6334.EFV57870 6334.EFV60001 0 0 0 0 0.506
0.156 0.264 0.228 0.731
CSRP2 EFV60002 6334.EFV57870 6334.EFV60002 0 0 0 0 0.506
0.156 0.264 0.228 0.731
CSRP2 tni-3 6334.EFV57870 6334.EFV61671 0 0 0 0 0.595 0.099
0.128 0.257 0.731
EFV47728 unc-15 6334.EFV47728 6334.EFV52403 0 0 0 0 0.139
0.138 0.221 0.158 0.448
EFV47728 mup-2 6334.EFV47728 6334.EFV56156 0 0 0 0 0.180 0
0 0.369 0.460
EFV47728 tni-3 6334.EFV47728 6334.EFV61671 0 0 0 0 0.175
0.118 0 0.343 0.480
EFV47728 LPP 6334.EFV47728 6334.EFV52047 0 0 0 0.725 0.113
0.157 0.329 0.467 0.521
EFV47728 SMTNL2 6334.EFV47728 6334.EFV61840 0 0 0 0 0.119
0.133 0.517 0.088 0.618
EFV47728 EFV60002 6334.EFV47728 6334.EFV60002 0 0 0 0
0.344 0.516 0.260 0.495 0.865
EFV47728 EFV60001 6334.EFV47728 6334.EFV60001 0 0 0 0
0.344 0.516 0.260 0.495 0.865
EFV48665 unc-15 6334.EFV48665 6334.EFV52403 0 0 0 0 0.139
0.138 0.221 0.158 0.448
EFV48665 mup-2 6334.EFV48665 6334.EFV56156 0 0 0 0 0.180 0
0 0.369 0.460
EFV48665 tni-3 6334.EFV48665 6334.EFV61671 0 0 0 0 0.175
0.118 0 0.343 0.480
EFV48665 LPP 6334.EFV48665 6334.EFV52047 0 0 0 0.719 0.113
0.157 0.329 0.467 0.522
EFV48665 SMTNL2 6334.EFV48665 6334.EFV61840 0 0 0 0 0.119
0.133 0.517 0.088 0.618
EFV48665 EFV60002 6334.EFV48665 6334.EFV60002 0 0 0 0
0.344 0.516 0.260 0.495 0.865
EFV48665 EFV60001 6334.EFV48665 6334.EFV60001 0 0 0 0
0.344 0.516 0.260 0.495 0.865
EFV55561 LPP 6334.EFV55561 6334.EFV52047 0 0 0 0.686 0.113
0.157 0.329 0.467 0.530
EFV55561 unc-15 6334.EFV55561 6334.EFV52403 0 0 0 0 0.139
0.138 0.221 0.158 0.448
EFV55561 mup-2 6334.EFV55561 6334.EFV56156 0 0 0 0 0.180 0
0 0.369 0.460
EFV55561 tni-3 6334.EFV55561 6334.EFV61671 0 0 0 0 0.175
0.118 0 0.343 0.480
EFV55561 SMTNL2 6334.EFV55561 6334.EFV61840 0 0 0 0 0.119
0.133 0.517 0.088 0.618
EFV55561 EFV60002 6334.EFV55561 6334.EFV60002 0 0 0 0
0.344 0.516 0.260 0.495 0.865
EFV55561 EFV60001 6334.EFV55561 6334.EFV60001 0 0 0 0
0.344 0.516 0.260 0.495 0.865
EFV60001 LPP 6334.EFV60001 6334.EFV52047 0 0 0 0 0.054
0.138 0.376 0.716 0.836
EFV60001 unc-15 6334.EFV60001 6334.EFV52403 0 0 0 0 0.624
0.208 0.240 0.266 0.811
EFV60001 mup-2 6334.EFV60001 6334.EFV56156 0 0 0 0 0.677
0.048 0.436 0.625 0.926
EFV60001 SMTNL2 6334.EFV60001 6334.EFV61840 0 0 0 0 0.045
0.137 0.479 0.097 0.560

EFV60001	EFV60002	6334.EFV60001	6334.EFV60002	0	0.617	0	0	0	0	0	0	0.617
EFV60001	tni-3	6334.EFV60001	6334.EFV61671	0	0	0	0	0.212	0.278	0.438	0.407	0.785
EFV60002	LPP	6334.EFV60002	6334.EFV52047	0	0	0	0	0.054	0.138	0.376	0.716	0.836
EFV60002	unc-15	6334.EFV60002	6334.EFV52403	0	0	0	0	0.624	0.208	0.240	0.266	0.811
EFV60002	mup-2	6334.EFV60002	6334.EFV56156	0	0	0	0	0.677	0.048	0.436	0.625	0.926
EFV60002	SMTNL2	6334.EFV60002	6334.EFV61840	0	0	0	0	0.045	0.137	0.479	0.097	0.560
EFV60002	tni-3	6334.EFV60002	6334.EFV61671	0	0	0	0	0.212	0.278	0.438	0.407	0.785
mup-2	unc-15	6334.EFV56156	6334.EFV52403	0	0	0	0	0.826	0.222	0.366	0.426	0.944
mup-2	tni-3	6334.EFV56156	6334.EFV61671	0	0	0	0	0.830	0.780	0.972	0.833	0.999
tni-3	unc-15	6334.EFV61671	6334.EFV52403	0	0	0	0	0.981	0.422	0.360	0.533	0.996
