# Supplementary figures and images for: Computational analysis of the flexibility in the disordered linker region connecting LIM domains in cysteine–glycine-rich protein
Source: Front Genet. 2023 Mar 29;14:1134509. doi: 10.3389/fgene.2023.1134509 (PMC10090389; doi:10.3389/fgene.2023.1134509)

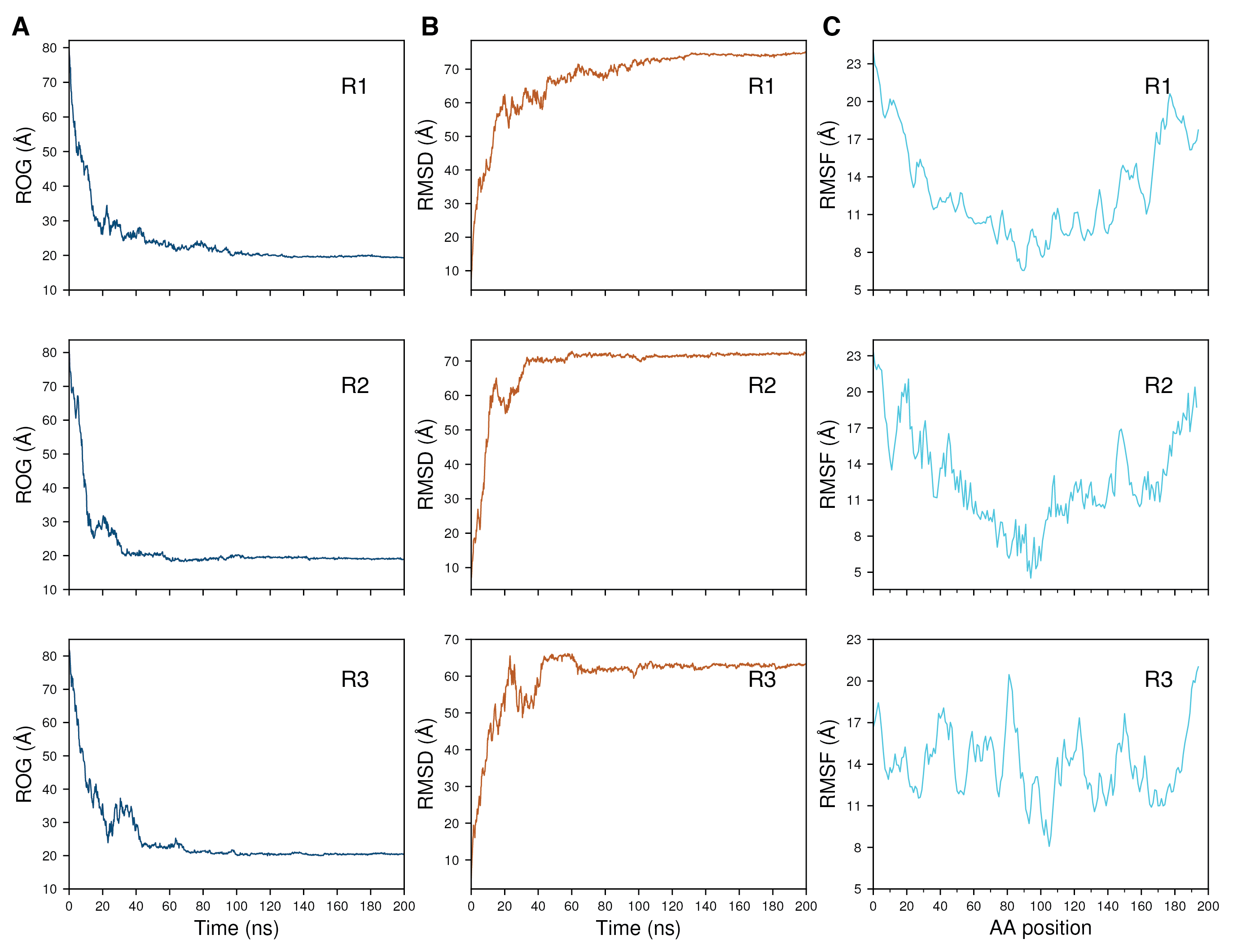

Supplement: Supplementary file 1 [file DataSheet1.zip › Supplementary_Figure_2.png]

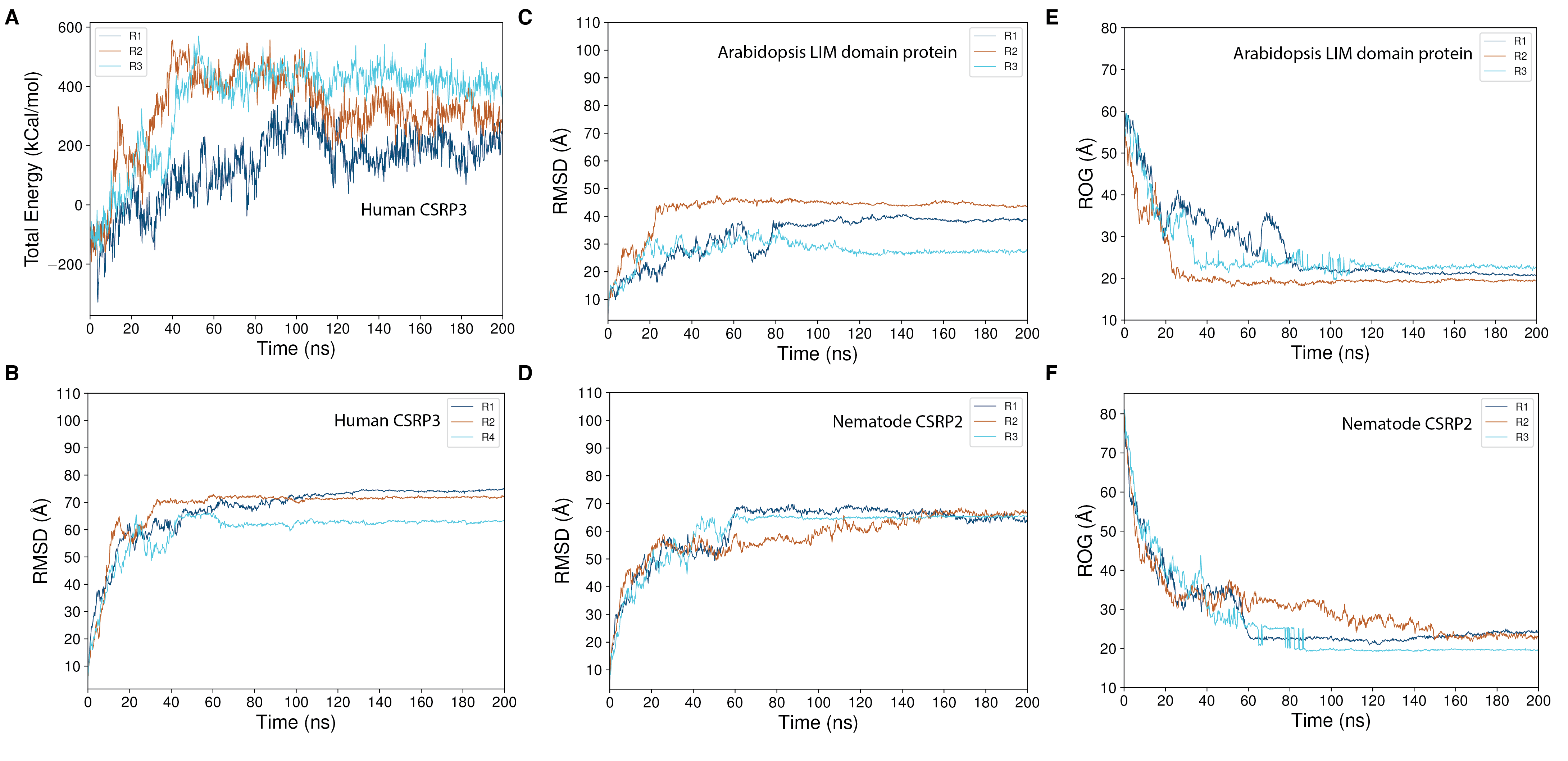

Supplement: Supplementary file 1 [file DataSheet1.zip › Supplementary_Figure_3.png]

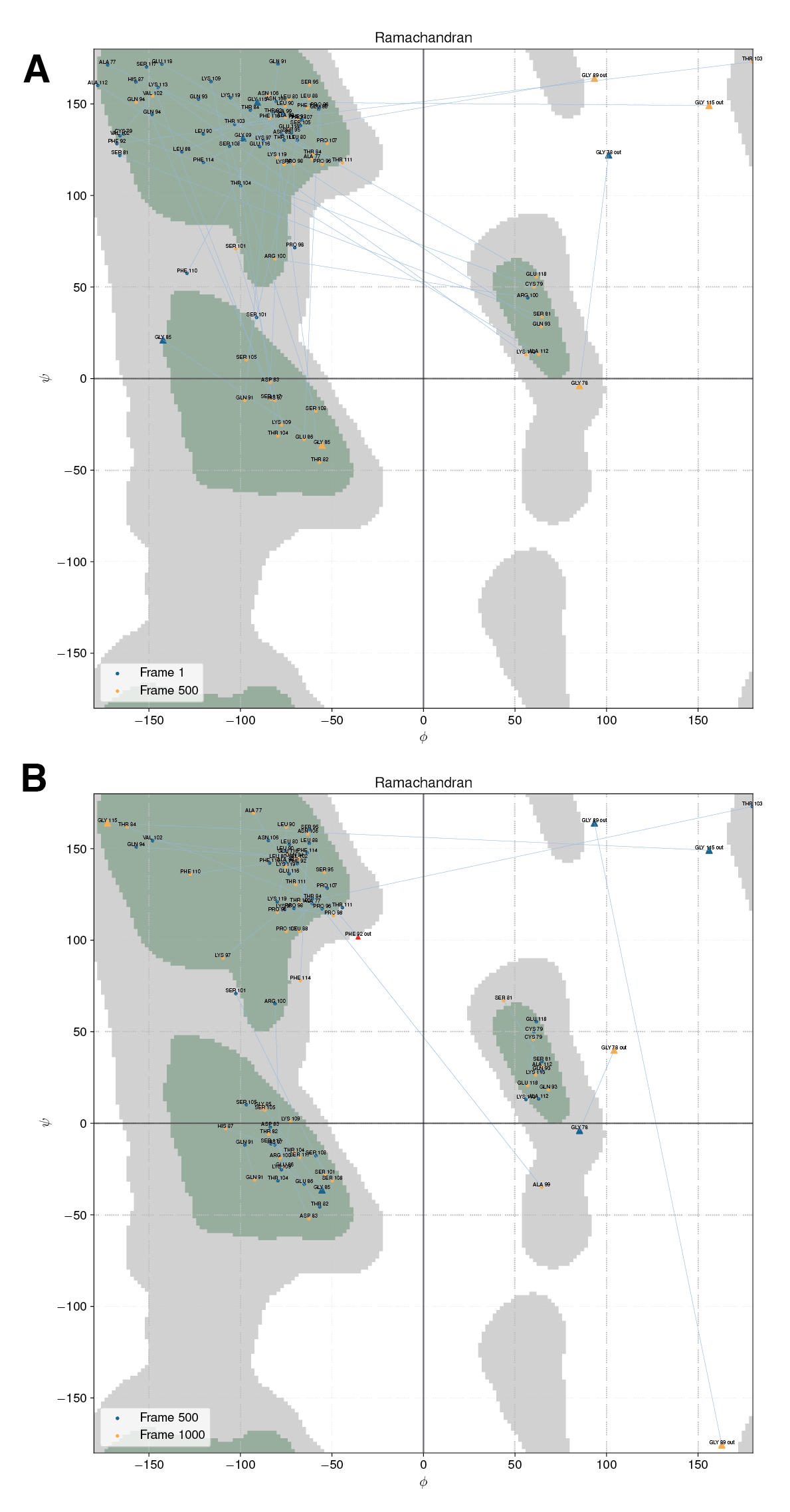

Supplement: Supplementary file 1 [file DataSheet1.zip › Supplementary_Figure_4.png]

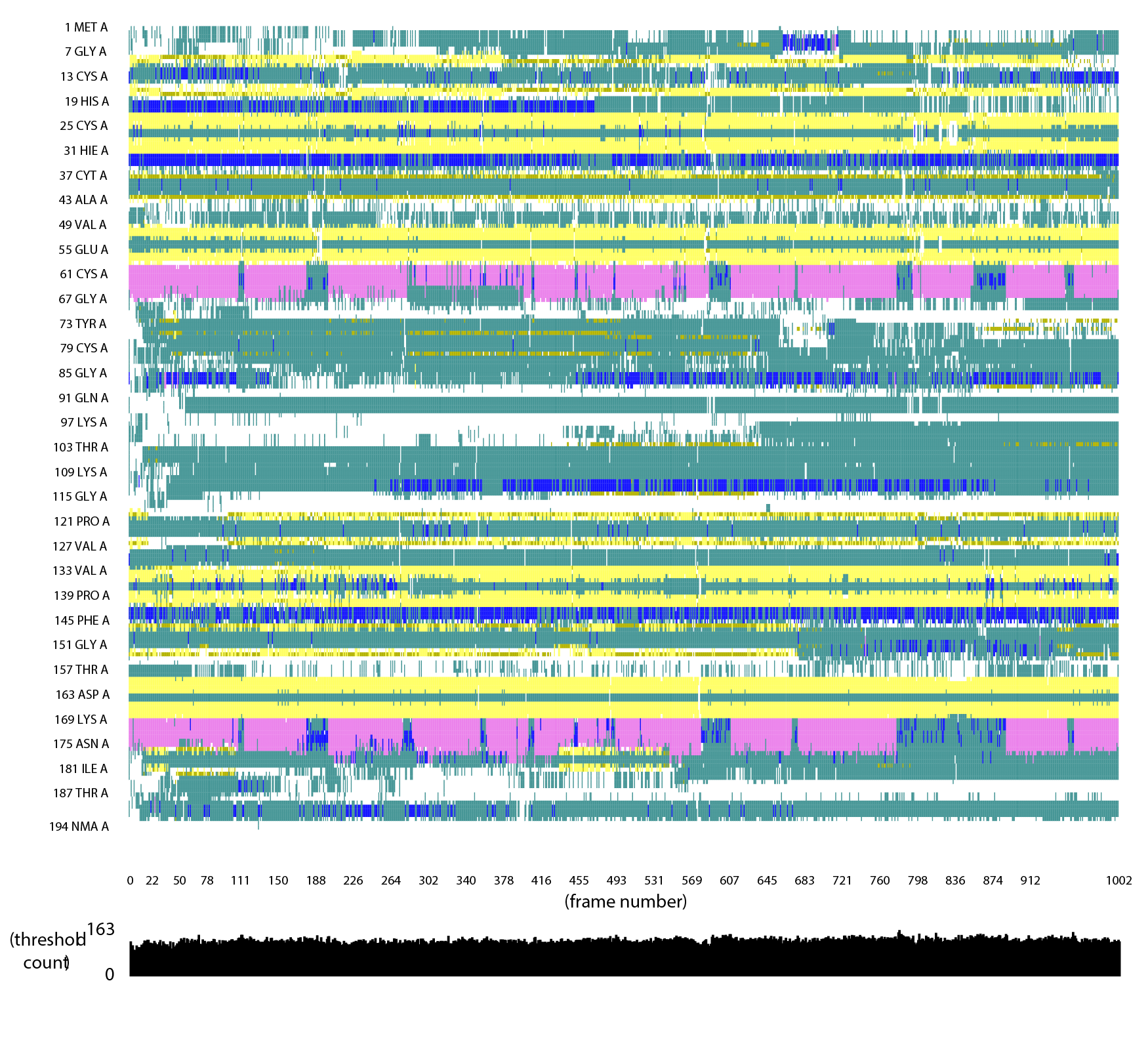

Supplement: Supplementary file 1 [file DataSheet1.zip › Supplementary_Figure_5.png]

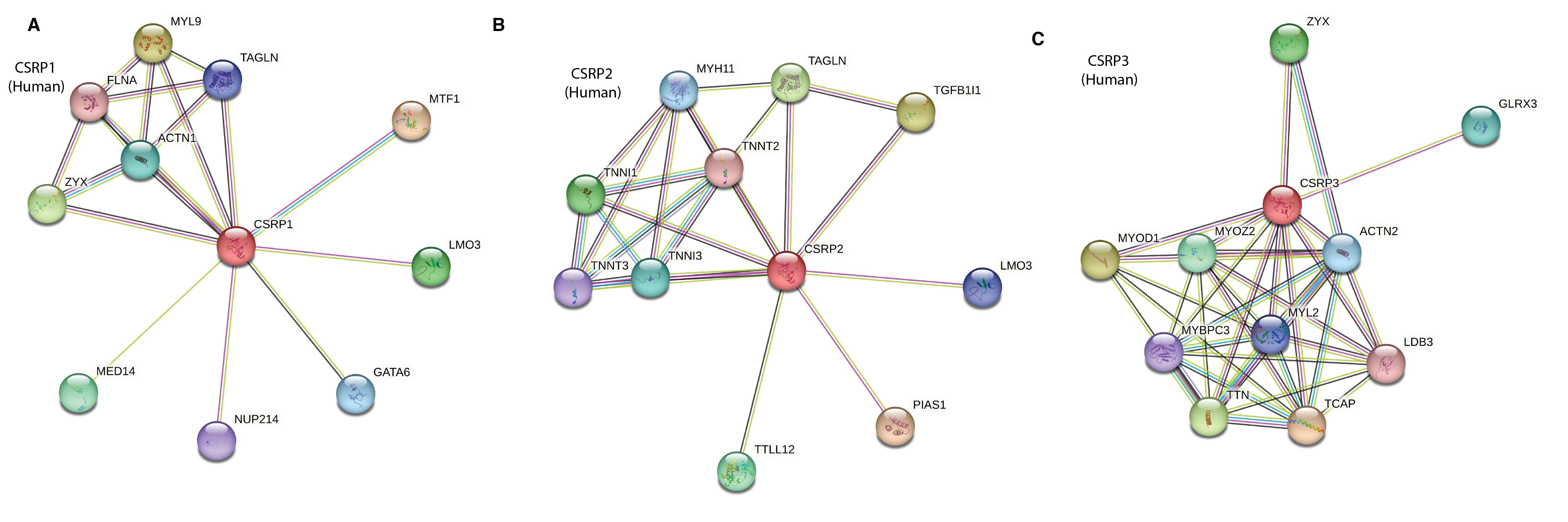

Supplement: Supplementary file 1 [file DataSheet1.zip › Supplementary_Figure_6.png]
