# Supplementary material for: Computational analysis of the flexibility in the disordered linker region connecting LIM domains in cysteine–glycine-rich protein
Source: Front Genet. 2023 Mar 29;14:1134509. doi: 10.3389/fgene.2023.1134509 (PMC10090389; doi:10.3389/fgene.2023.1134509)

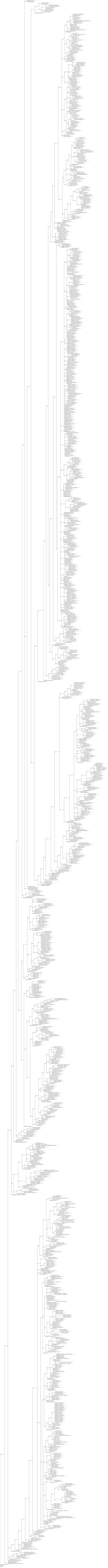

Supplement: Supplementary file 1 [file Supplementary_Figure_1.pdf › Supplementary_Figure_1.pdf]
